# Single cell sequencing maps skeletal muscle cellular diversity as disease severity increases in dystrophic mouse models

**DOI:** 10.1016/j.isci.2022.105415

**Published:** 2022-10-21

**Authors:** Kholoud K. Saleh, Haibin Xi, Corey Switzler, Emily Skuratovsky, Matthew A. Romero, Peggie Chien, Devin Gibbs, Lily Gane, Michael R. Hicks, Melissa J. Spencer, April D. Pyle

**Affiliations:** 1 Department of Molecular, Cellular and Integrative Physiology, University of California Los Angeles, Los Angeles, CA 90095, USA; 2 Department of Microbiology, Immunology, and Molecular Genetics, University of California Los Angeles, Los Angeles, CA 90095, USA; 3 Eli and Edythe Broad Center of Regenerative Medicine and Stem Cell Research, University of California Los Angeles, Los Angeles, CA 90095, USA; 4 CIRM Bridges Program, California State University, Northridge, CA 91330, USA; 5 Department of Neurology, University of California Los Angeles, CA 90095, USA; 6 Molecular Biology Institute, University of California Los Angeles, Los Angeles, CA 90095, USA

**Keywords:** Animal physiology, Biological sciences, Cellular physiology, Natural sciences, Omics, Physiology, Transcriptomics

## Abstract

Duchenne muscular dystrophy (DMD) is caused by out-of-frame mutations in the DMD gene resulting in the absence of a functional dystrophin protein, leading to a devastating and progressive lethal muscle-wasting disease. Little is known about cellular heterogeneity as disease severity increases. Advances in single-cell RNA sequencing (scRNA-seq) enabled us to explore skeletal muscle-resident cell populations in healthy, dystrophic, and severely dystrophic mouse models. We found increased frequencies of activated fibroblasts, fibro-adipogenic progenitor cells, and pro-inflammatory macrophages in dystrophic gastrocnemius muscles and an upregulation of extracellular matrix genes on endothelial cells in dystrophic and severely dystrophic muscles. We observed a pronounced risk of clotting, especially in the severely dystrophic mice with increased expression of plasminogen activator inhibitor-1 in endothelial cells, indicating endothelial cell impairment as disease severity increases. This work extends our understanding of the severe nature of DMD which should be considered when developing single or combinatorial approaches for DMD.

## Highlights

• scRNA-seq reveals cell differences in healthy, dystrophic, and severely dystrophic muscles
• Increased frequency of activated fibroblasts and FAP cells in dystrophic environments
• Co-existence of pro- and anti-inflammatory signatures in dystrophic environments
• Endothelial cell impairment is evident in severely dystrophic environment

## Graphical abstract

**Figure:**
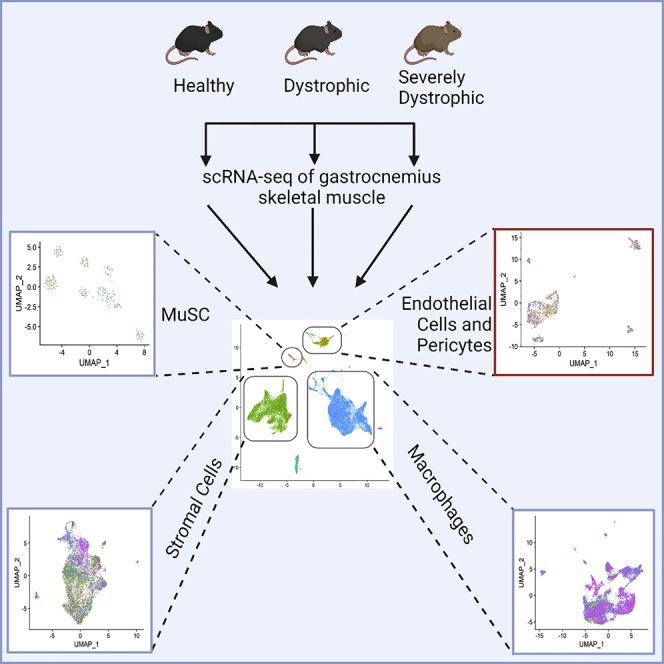

## Introduction

Duchenne muscular dystrophy (DMD) is an X-linked recessive, severe progressive muscle wasting disease affecting ∼1–5000 male live births (Mendell and Lloyd-Puryear, 2013; Mendell et al., 2012; Moat et al., 2013). Both devastating and fatal, DMD patients are diagnosed typically before their fifth year of age, are wheelchair bound in their teens, and prematurely die in their thirties. DMD is caused by a loss-of-function mutation in the *DMD* gene, the largest known gene in the human genome, that results in the absence of a functional dystrophin protein (Hoffman et al., 1987). The regenerative capacity of skeletal muscle is mainly achieved through the differentiation of satellite cells, a muscle stem cell (MuSC) population present between the basal lamina and sarcolemma of the muscle fibers (Lepper et al., 2011; Mauro, 1961; Murphy et al., 2011; Sambasivan et al., 2011). In DMD, impaired regeneration arises because either MuSCs are rendered dysfunctional because of impaired polarity establishment or because of progressive exhaustion (Dumont et al., 2015; Sacco et al., 2010; Webster and Blau, 1990). Eventually, lack of proper regeneration leads to muscle fiber necrosis and generation of excess fibrotic tissue (Klingler et al., 2012).

The most common mouse model used to study DMD is the mdx mouse, which has a nonsense mutation in exon 23 in the X chromosome that arose spontaneously in a C57BL/10 colony (Bulfield et al., 1984; Sicinski et al., 1989). The mdx mouse model lacks dystrophin protein, has elevated plasma levels of muscle creatine kinase, and present histological muscle lesions like that of human disease. However, adult mdx mice do not fully recapitulate the human disease in terms of pathogenic progression. The mdx mouse model lifespan is not significantly reduced, regeneration of muscle fibers is not persistent, and lacks extensive fibro-fatty replacement of muscle fibers, thus the mdx model does not fully recapitulate human clinical disease progression (Carnwath and Shotton, 1987; COULTON et al., 1988; DiMario et al., 1991). Because the discovery of mdx mice, several other mouse models have been generated for DMD in different genetic backgrounds. One of which is the DBA/2-mdx (hereafter referred to as mdxD2) mouse strain. MdxD2 mice exhibit lower muscle weight, fewer muscle fibers and increased fibro-fatty deposition in comparison with the mdx strain (Fukada et al., 2010). Genetic modifiers on the DBA/2 genetic background, including osteopontin (Spp1), synonymous variant in Annexin A6 (Anxa6) exon 1, and polymorphisms in the coding region of the latent TGF-β-binding protein 4 gene (Ltbp4), generate a more severe muscular dystrophy mouse model (Heydemann et al., 2009; Quattrocelli et al., 2017; Swaggart et al., 2014). The exacerbated TGF-β signaling, increased inflammation, increased fibrosis, and progressive weakness and atrophy in the mdxD2 mouse model better recapitulate the characteristics of DMD human disease (Capote et al., 2016; Hammers et al., 2020; Mázala et al., 2020).

Recently, single-cell RNA sequencing (scRNA-seq) has improved our understanding of skeletal muscle and the cellular dynamics and myogenic continuum of homeostatic and regenerating muscle (Cho and Doles, 2017; Dell’Orso et al., 2019; Giordani et al., 2019; Micheli et al., 2020; Oprescu et al., 2020). Recent studies have shown that endothelial cells (ECs) are incredibly heterogeneous in healthy muscle (Fan et al., 2021; Kalucka et al., 2020; Tombor et al., 2021). In addition, the cross-talk between ECs and MuSCs has been established (Christov et al., 2007; Verma et al., 2018,^2021^). However, a thorough characterization of skeletal muscle-resident cellular composition and dynamics in pathological conditions, such as in DMD mouse models, has not been described at the single-cell transcriptomic level. Moreover, in DMD mouse models there is little understanding of the cellular interaction between supportive cells in the muscle, such as stromal cells and macrophages, and ECs.

Here, we evaluated the cellular composition of skeletal muscle-resident cell populations between healthy (wt-NSG), dystrophic (mdx-NSG), and severely dystrophic (mdxD2-NSG) mouse models, which are used routinely to evaluate cell-based therapeutics. We found that as the disease severity increases, the dynamics of cellular subpopulations of stromal cells, macrophages, and ECs changes. We demonstrate an increased prevalence of macrophages, both pro-inflammatory and anti-inflammatory macrophages, activated fibro-adipogenic progenitor (FAP) cells and, activated fibroblasts in the dystrophic and severely dystrophic muscles. Moreover, we identify a capillary EC subpopulation with an increased occurrence in severely dystrophic muscle. To further our understanding of EC differences between healthy and DMD conditions, and the role that the microenvironment plays in such differences, we investigated interactions between predicted released ligands from stromal cells and macrophages, and their potential differentially expressed target genes on EC, utilizing NicheNet analysis (Browaeys et al., 2020). To the best of our knowledge, for the first time we have identified upregulation of extracellular matrix (ECM) genes in severely dystrophic ECs. We have further identified the upregulation of plasminogen activator inhibitor-1 (PAI-1), a serine protease inhibitor that functions as procoagulant, in severely dystrophic ECs, indicating further functional impairment of ECs (Handt et al., 1996). Overall, this work provides a resource for understanding how the cellular dynamics and skeletal muscle microenvironment changes as disease severity progress and could provide potential avenues for considering when developing combination stem cell or single therapeutics for DMD.

## Results

### scRNA-seq reveals skeletal muscle cellular heterogeneity between healthy and DMD immunodeficient mouse models

**Figure 1:**
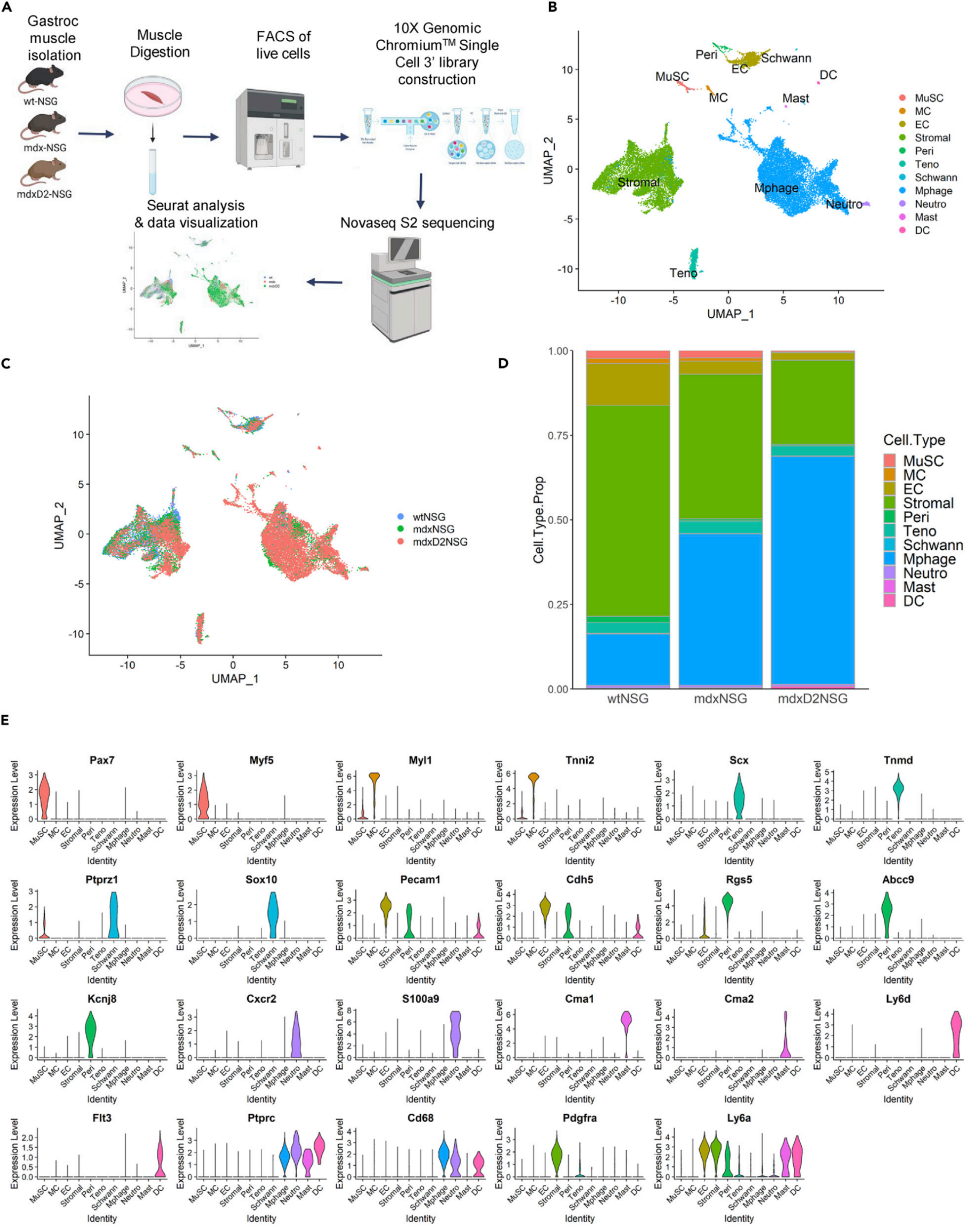
Single-cell RNA sequencing reveals cellular heterogeneity between healthy and dystrophic muscle states (A) Schematic illustrating the experimental workflow for scRNA-seq with 10X genomics platform. Briefly, single-cell suspension from right gastrocnemius muscles was prepared by mechanical and enzymatic dissociation followed by live cell sorting. Following library preparation, sequencing was performed on Illumina Novaseq 6000 S2 platform, and the raw data were processed by Cell Ranger (10X Genomics) to generate a gene-cell expression matrix. Metrices were then analyzed individually using Seurat and integrated using Harmony packages (n = 2 replicates for each mouse model). (B) UMAP embedding of integrated scRNA-seq data with a total of 20,920 cells analyzed from wt-NSG, mdx-NSG and mdxD2-NSG gastrocnemius muscles (See also Table S1 for cell population counts in each mouse model). (C) UMAP embedding of integrated scRNA-seq data grouped by mouse model. (D) Relative proportion of cell populations in healthy, dystrophic and severely dystrophic gastrocnemius skeletal muscles. (E) Violin Plot showing the main gene expression markers used to identify gastrocnemius skeletal muscle cellular composition (also see Table S2 for top 20 marker genes in each cluster). MuSC: muscle satellite cells, MC: myocytes, EC: endothelial cells, Stromal: stromal cells including fibro-adipogenic progenitor cells and fibroblasts, Peri: pericytes, Teno: tenocytes, Schwann: Schwann cells, Mphage: macrophages, Neutro: neutrophils, Mast: Mast cells, DC: dendritic cells.

Using scRNA-seq we evaluated changes in cellular composition of 8-week-old healthy (wt-NSG), dystrophic (mdx-NSG) and severely dystrophic (mdxD2-NSG) gastrocnemius muscles. In brief, single-cell suspensions from gastrocnemius muscles were prepared, live cells were collected using fluorescence-activated cell sorting (FACS), the single cell libraries were generated using the 10X Genomics Chromium platform and samples were sequenced on Illumina NovaSeq 6000 platform (n = 2 muscles per mouse model, individually sorted and sequenced) (Figure 1A). Samples were first individually analyzed using Seurat by performing filtering, normalizing, scaling, and dimensionality reduction analysis (Figures S1A–S1C). After the initial analysis we were able to obtain and integrate a total of 4180, 5723 and 11,017 cells from wt-NSG, mdx-NSG and mdxD2-NSG, respectively, using the Harmony package (Figures S1D and S1E; Table S1) (Korsunsky et al., 2019). We used uniform manifold approximation and projection (UMAP) to visualize our scRNA-seq dataset (Figures 1B and 1C)(Korsunsky et al., 2019). Unsupervised clustering resulted in a total of 46 different clusters in our integrated data which we merged based on gene expression into established identified cell types in skeletal muscle.

Muscle satellite cells (MuSC) were marked by *Pax7* and *Myf5* expression, myocytes (MC) were marked by *Myl1* and *Tnni2*, tenocytes (Teno) were marked by *Scx* and *Tnmd*, Schwann cells (Schwann) were marked by *Ptprz1* and *Sox10*, ECs were marked by *Pecam1* and *Cdh5* and pericyte (Peri) expressed *Rgs5, Abcc9*, and *Kcnj8* (Bondjers et al., 2003,^2006^; Giordani et al., 2019; Micheli et al., 2020; Xi et al., 2020). Because these mice are immunodeficient, they lack mature T-, B- and natural killer cells which are absent in our scRNA-seq set. However, we were able to detect clusters of immune cells namely neutrophils (Neutro), which are marked by *Cxcr2* and *S100a9*, mast cells (Mast), marked by *Cma1* and *Cma2*, and dendritic cells (DC), marked by *Ly6d* and *Flt3*, which are not ablated in NSG immunodeficient mouse models. In addition, we were able to capture macrophages, marked by *Ptprc* and *Cd68*, which constituted one of the largest clusters in mdx-NSG and mdxD2-NSG muscles (Figure 1D). Stromal cells (Stromal), marked by *Pdgfrα* and *Ly6a,* constituted the largest cluster in wt-NSG muscles, and second largest clusters in mdx-NSG and mdxD2-NSG muscles. Gene expression markers used to identify each cell type cluster is shown in Figure 1E and a list of the top 20 marker genes in each cell type cluster is provided in Table S2 (Table S2, relevant to main Figure 1E).

### Muscle satellite cell sub-clustering reveals differences in stem cell states

**Figure 2:**
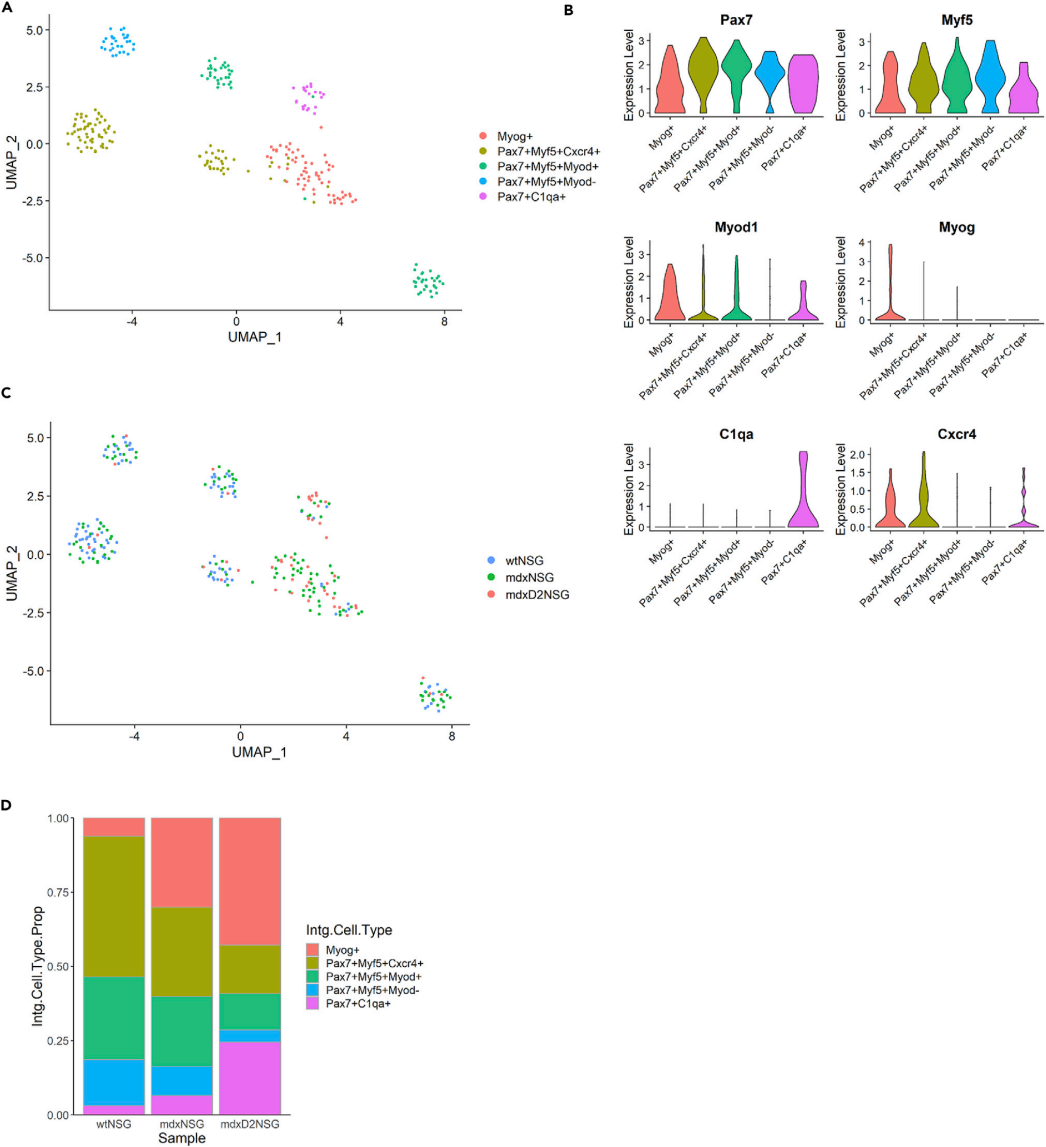
Muscle satellite cell sub-clustering reveals differences in stem cell states (A) UMAP embedding of integrated muscle stem cell subpopulations in all mouse models. (B) UMAP embedding representing mouse model contribution to integrated muscle stem cell subpopulations. (C) Gene expression of markers used to identify the muscle stem cell subpopulations in integrated UMAP. (D) Relative proportion of muscle stem cell subpopulations in each mouse model.

To explore the transcriptional changes between healthy, dystrophic and severely dystrophic MuSC, we subclustered a total of 269 MuSCs, performing further normalization and integration of MuSCs to identify cellular subtypes. Unsupervised clustering resulted in a total of 6 subpopulations in which we merged to 5 MuSC subtypes or derivatives based on their gene expression profile (Figure 2). We identified a cluster of differentiated or committed MuSC was marked by *Myog* (Myog+), and a cluster of quiescent satellite cells that was marked by *Pax7*, *Myf5*, *Chodl* and *Spry1* (Pax7+Myf5+Myod1-). Moreover, we identified two clusters of activated MuSC with *Pax7*, *Myf5* and *Myod1* expression, however one of these clusters only was enriched for Cxcr4 (Pax7+Myf5+Cxcr4+, and Pax7+Myf5+Myod+) (Figures 2A and 2B). Of interest, we have identified a MuSC cluster that had an immune signature expressing *C1qa/b*, *Lyz2* and *Cd53* (Figure 2A). This immune cluster comprised about 25% of MuSCs subtypes in mdxD2-NSG model (Figures 2C and 2D). A cluster of immune signature was identified recently in regenerating muscle where 21 days post injury about 50% of MuSC were identified as immuno-myoblast (Oprescu et al., 2020). Collectively, our findings show differences in the MuSC cellular subtype compositions and states between healthy and dystrophic muscles, where it is clearly evident that the quiescent MuSCs consists of smaller proportion of the total MuSCs in dystrophic environments, whereas a higher proportion of committed and differentiated MuSCs are evident as disease severity increases. We further identified a MuSC subpopulation with an immune signature that is highly expressed/present in severely dystrophic muscle.

### Stromal cell sub-clustering reveals stromal subtypes arising in dystrophic and severely dystrophic muscle

**Figure 3:**
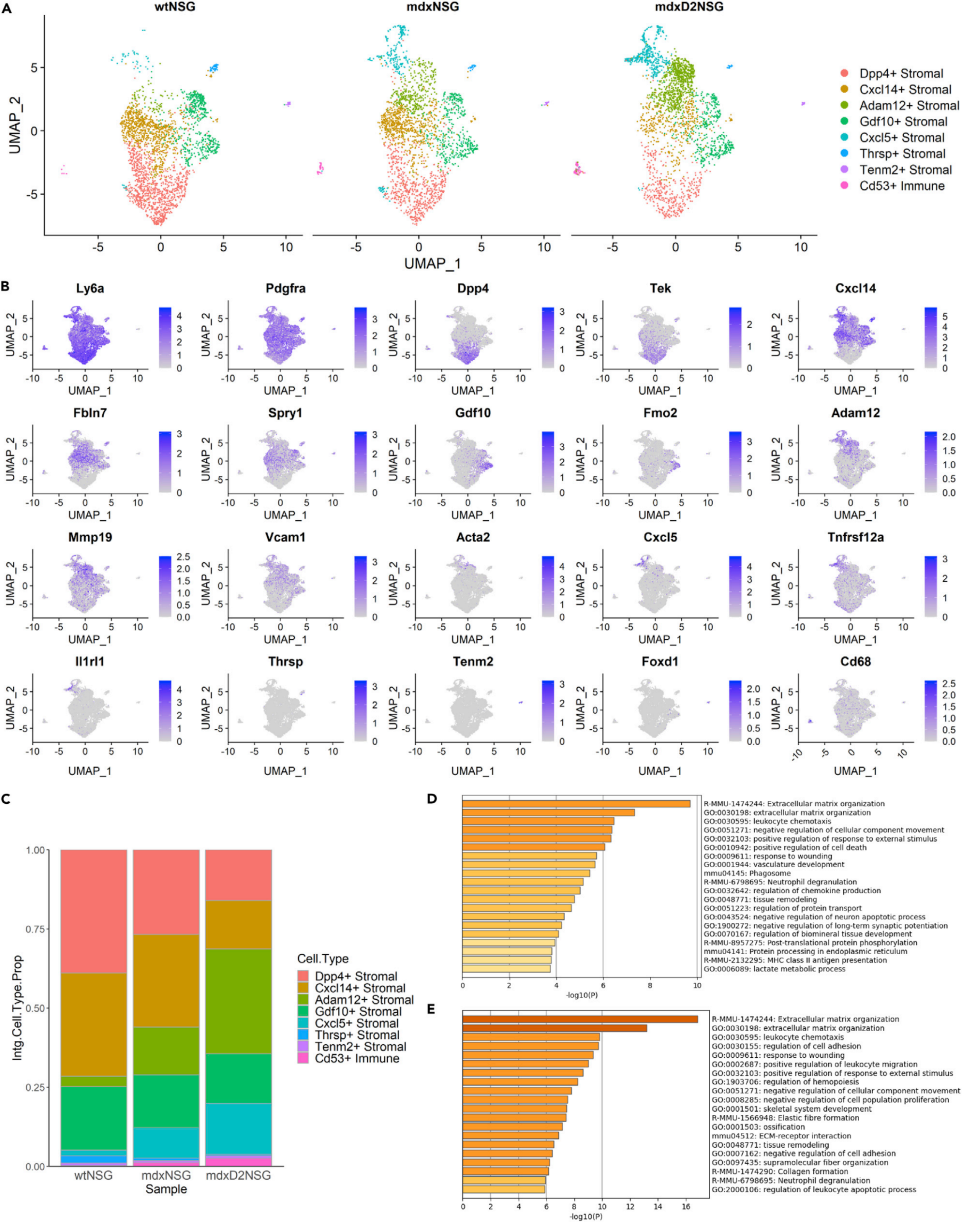
Stromal cell sub-clustering reveals stromal subtypes arising in severely dystrophic muscle (A) UMAP of stromal cell subpopulations split by mouse model. (B) Marker gene expression used to identify stromal cell subpopulations in integrated UMAP. (C) Relative proportion of stromal cell subpopulations in healthy, dystrophic and severely dystrophic muscles. (D) GO enrichment analysis of DE-Gs in mdxD2-NSG stromal cells versus mdx-NSG. (E) GO enrichment analysis of DE-Gs in mdxD2-NSG stromal cells versus wt-NSG.

One of the most striking differences that we observed in cellular composition between healthy, dystrophic and severely dystrophic skeletal muscles was the shift of the stromal cell population percentages, from being the most abundant cell type in healthy gastrocnemius muscle of wt-NSG mice, accounting for 62% of all cells, to lower proportions in dystrophic muscles at 42% in mdx-NSG and only 25% of all cells in mdxD2-NSG (Figure 1D). To discriminate the different stromal cell subtypes present, we subclustered a total of 7526 cells and performed differential gene expression within the cluster, performing further normalization and integration of the subset data (Figure 3). We were able to identify seven stromal cell subtypes and one subtype with immune identity (Figure 3A). The gene expression profile of the integrated data used to name and identify the clusters is shown in Figure 2B and an expanded panel of gene expression marker list is shown in Figure S2. *Dpp4*+ Stromal cluster expressed *Dpp4* and *Tek*, and the *Cxcl14*+ Stromal cluster expressed *Cxcl14*, *Fbln7* and *Spry1. Dpp4+*Stromal and *Cxcl14+*Stromal*,* resembled FAPs which were similarly identified in non-injured tibialis anterior muscle (Oprescu et al., 2020). The *Dpp4+*stromal cell subtype has a similar gene expression signature to Tie2^high^Vcam1^low^ identified in C57Bl/10 WT model (Malecova et al., 2018). Of interest, the proportion of these stromal cell subtypes decreases significantly in severely dystrophic environment falling from a total of 72% in healthy muscle, to 56% in dystrophic muscle, to only 31% in severely dystrophic muscle (Figure 3C). *Adam12+*Stromal cell subtype, which appear to be activated fibroblast, expressed *Adam12, Mmp19, Vcam1 and Acta2* and high levels of *Postn* (Fu et al., 2018) (Figure S2A). In mdxD2-NSG muscle, *Adam12+*stromal cell subpopulation constituted about 33% of the total stromal cell composition making it the dominant stromal cell subpopulation, whereas only 15 and 3% of stromal cell composition in mdx-NSG and wt-NSG muscles, respectively. *Cxcl5*+ Stromal cell subtype, which appear to be activated FAPs, was marked by the expression of *Cxcl5*, *Tnfrsf12a*, and *Il1rl1*. The *Cxcl5+*Stromal has been also described in an acute injury model (Micheli et al., 2020; Oprescu et al., 2020), and in our dataset its proportion increases from about 2% in healthy wt-NSG muscle, to about 10% in dystrophic mdx-NSG, and 16% in severely dystrophic mdxD2-NSG environments (Figure 3C). Both *Adam12+*and *Cxcl5+*Stromal subtypes have a Tie2^low^Vcam1+ signature similar to published work in the mdx model, which emphasizes the power of scRNA-seq in further deconvoluting the cellular composition of skeletal muscle cell types (Malecova et al., 2018) (Figure S2B).

In addition, we were able to identify a stromal cell subtype of traditional fibroblasts marked by *Gdf10* and *Fmo2* expression, labeled *Gdf10*+ Stromal. *Gdf10*+ Stromal has been described to inhibit adipogenesis and was shown to decrease in dystrophic muscles of Sarcoglycan-null mouse model (Camps et al., 2020). In our dataset, the proportion of *Gdf10+*Stromal subtype decreased from 20% in healthy wt-NSG muscle, to about 17% in dystrophic, to about 16% in severely dystrophic mdxD2-NSG muscle. We have, moreover, identified three small stromal subtypes that clustered separately from other stromal cells and had a distinct gene expression profile. These were identified as *Thrsp+*Stromal*,* labeled by *Thrsp* expression, *Tenm2+*Stromal, labeled by *Tenm2* and *Foxd1* expression, and *Cd53+*Immune, labeled by *Cd53* and *Cd68* expression. Although the *Cd53+*Immune cell subtype expresses *Ly6a* and *Pdgfra*, it also has an immune profile including expression of *Cd68* and *Cd53*. Overall, we observed stromal cell subtype shifts with a reduction in FAPs, a reduction in traditional fibroblasts and an increase in activated fibroblasts in mdxD2-NSG compared to both wt-NSG and mdx-NSG.

We further examined the differentially expressed genes (DE-Gs) upregulated in the total stromal cell cluster in mdxD2-NSG versus wt-NSG and mdx-NSG, followed by Gene Ontology (GO) enrichment analysis to identify enriched biological processes and signaling pathways (Figures 3D and 3E) (Zhou et al., 2019). The main GO enriched term was extracellular matrix (ECM) organization, with an enrichment of genes such as *Fn1*, *Fbn2*, *Fbln1,* and collagen genes such as *Col8a1*, *Col14a1* and *Col16a1* between mdxD2-NSG and wt-NSG, and *Adam12*, *Col12a1*, *Col14a1*, *Col8a1* and *Plod2* enriched in mdxD2-NSG compared to mdx-NSG. Of interest, the GO enriched term for regulation of chemokine production was observed in mdxD2-NSG compared to mdx-NSG, with enriched genes such as *Il1r1*, *Postn, Ccl2*, *Ccl6*, *Cxcl12*, and *Cxcl5.* Overall, our findings helped identify stromal cell dynamics in healthy and muscular dystrophy states. Our scRNA-seq dataset provide a powerful tool that could further untangle stromal cells contribution to the severity of muscular dystrophy.

### Macrophage heterogeneity between healthy, dystrophic and severely dystrophic skeletal muscle

**Figure 4:**
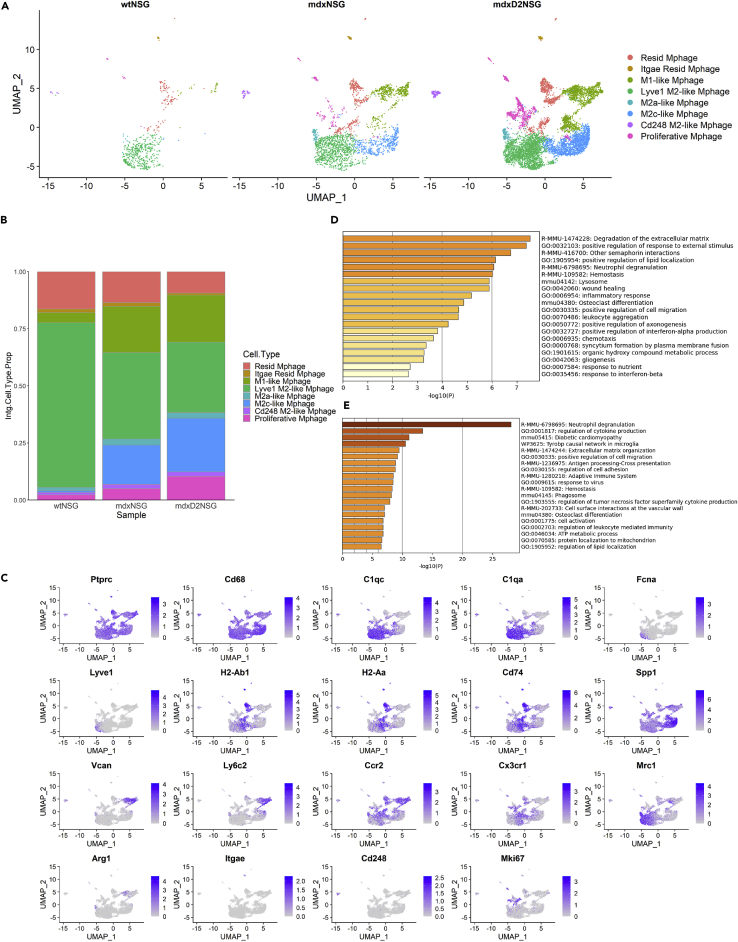
Macrophage heterogeneity between healthy, dystrophic and severely dystrophic skeletal muscle (A) UMAP embedding of macrophage subpopulations gastrocnemius skeletal muscle split by mouse model. (B) Relative proportion of macrophage subpopulations cellular composition between mouse models. (C) Gene expression panel of markers used to distinguish the different macrophage subpopulations in integrated UMAP. (D) GO enrichment analysis of DE-Gs in all mdxD2-NSG macrophages versus mdx-NSG. (E) GO enrichment analysis of DE-Gs in all mdxD2-NSG macrophages versus wt-NSG. Resid Mphage = Resident macrophages.

Next, we explored macrophage heterogeneity because it is well known that macrophages play pivotal roles in exacerbating muscle pathology in dystrophic muscles (Capote et al., 2016; Rigamonti et al., 2014; Tidball et al., 2018; Villalta et al., 2009; Wehling et al., 2001). In our scRNA-seq dataset the frequency of macrophage cluster increases from 15% in wt-NSG, 639 cells, to 44%, 2560 cells, and 67%, 7426 cells, in mdx-NSG and mdxD2-NSG, respectively (Figure 1D). To further our understanding of macrophage population heterogeneity, we subclustered the macrophages (Mphage) and performed further normalization and integration of the subset data (Figure 4). Unsupervised clustering resulted in a total of ten different macrophage subtypes, which we merged into classically and alternatively activated macrophage clusters depending on their gene expression profile (Figure 4A). Compared to dystrophic muscles, macrophage heterogeneity was less evident in healthy muscle with M2a-like Mphage and M2c-like Mphage subpopulations arising in mdx-NSG and mdxD2-NSG muscles (Figure 4B). Gene expression profiles of markers used to identify the macrophage subtypes are shown in Figure 4C. Resident macrophage subtype, marked by *H2-Ab1*, *H2-Aa*, and *Cd74*, and *Lyve1*+ M2-like macrophages, marked by *C1qc*, *C1qa*, *Fcna* and *Lyve1*, were the dominant subtypes of macrophage cellular composition in wt-NSG, accounting for a total of about 88%, whereas it accounted for a total of 52% of macrophages cellular composition in dystrophic mdx-NSG muscle, and only 40% in severely dystrophic mdxD2-NSG muscle. M1-like macrophage cluster was mainly evident in dystrophic mdx-NSG muscle, constituting a proportion of about 20%, and severely dystrophic muscle with about 21% macrophage composition, whereas it accounted for only 4% of healthy muscle macrophage subtypes. We identified this cluster by the expression of *Ly6c2, Ccr2, Arg1, Vcan* and low expression of *Cx3cr1* (Figure 4C). This cluster has been described previously in the mdx muscle to exert pro-fibrotic functions toward fibroblasts (Juban et al., 2018). M2c-like macrophages, identified by the expression of *Spp1*, is another predominant macrophage subtype in mdx-NSG muscle, constituting about 17%, and mdxD2-NSG muscle, at about 23% of total macrophage cellular composition, whereas it constituted less than 1% in healthy muscle macrophage subtype composition. M2a-like macrophage cluster, marked by the expression of *Mrc1* and *Arg1*, is detected comprising about 3 and 2% in mdx-NSG and mdxD2-NSG of total macrophages compared to about 1% in wt-NSG muscle. Other small macrophage subpopulations that clustered separately were identified as Cd248 M2-like, marked by the expression of *Cd248*, Itgae resident macrophages, marked by the expression of both *Cd74* and *Itgae*, and proliferative macrophages, marked by the expression of *Mki67*. These findings indicate the complexity of macrophage subpopulations in both dystrophic and severely dystrophic environments and the involvement of dynamic macrophage phenotypes in the pathological process of muscular dystrophy (Singh and Chazaud, 2021). GO analysis of DE-Gs in mdxD2-NSG macrophages showed enriched terms for degradation of the ECM*,* positive regulation of lipid localization, *Abcg1, Plin2, Lpl, Trem2, Spp1* and *Mif1,* regulation of cytokine production, with upregulated genes such as *Acp5, Arg1, Stat1, Mmp12* and *Pld3,* among others (Figures 4D and 4E).

Overall, our findings provide a tool to explore the dynamic existence of pro-inflammatory, M1, and anti-inflammatory, M2, macrophages in the dystrophic and severely dystrophic muscle. Moreover, it provides insights on the involvement of immune cells in the severity of muscular dystrophy with their increased relative proportion compared to dystrophic and healthy skeletal muscles. Our scRNA-seq macrophage dataset and our integration method detected small populations of macrophages that need to be further studied to elucidate their pathological contribution to muscular dystrophy disease severity.

### Characterization of endothelial cell subpopulations in healthy, dystrophic and severely dystrophic skeletal muscle

**Figure 5:**
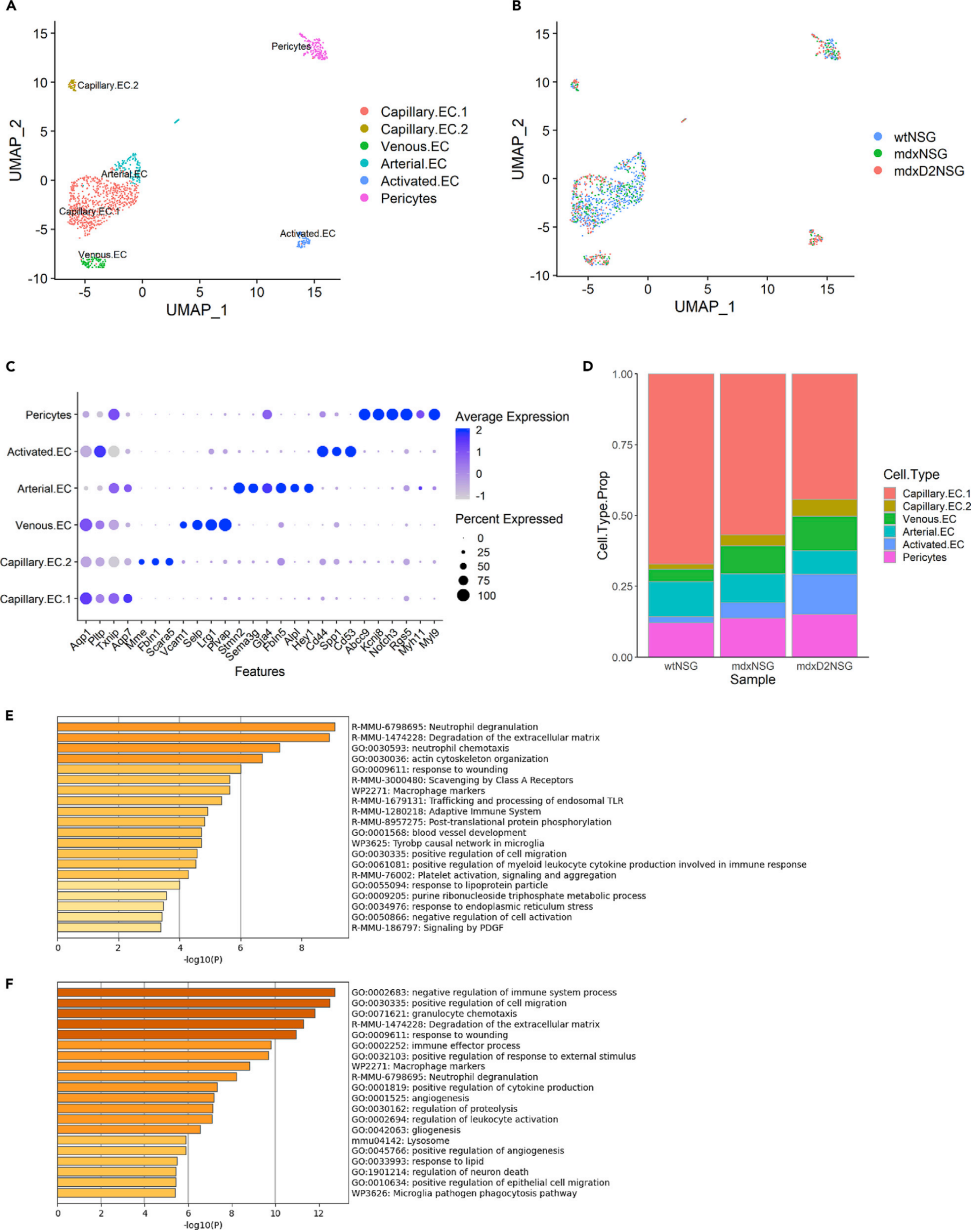
Characterization of endothelial cell subpopulations in healthy, dystrophic and severely dystrophic skeletal muscle (A) UMAP embedding of integrated pericytes and endothelial cell subpopulations. (B) UMAP embedding representing mouse model contribution to endothelial cell subpopulations. (C) Dot plot of chosen genes used to identify subtypes of endothelial cells (see also Table S3 for top 20 genes expressed in each endothelial cell subpopulation cluster). (D) Relative proportion of endothelial cell subpopulations identified in healthy and dystrophic muscles. (E) GO enrichment analysis of DE-Gs in mdxD2-NSG endothelial cells versus mdx-NSG. (F) GO enrichment analysis of DE-Gs in mdxD2-NSG endothelial cells versus wt-NSG.

An often unexplored cell population in published scRNA-seq datasets is the EC population, although its critical role in maintaining skeletal muscle homeostasis and in regeneration has been demonstrated by multiple groups (Christov et al., 2007; Shimizu-Motohashi and Asakura, 2014; Verma et al., 2018,^2019^). To examine EC differences between healthy, dystrophic, and severely dystrophic muscles, a total of 1150 ECs and pericytes were analyzed by performing differential gene expression within these clusters (Figures 5A and 5B). We used genes enriched in each EC subtype to annotate the clusters based on published literature (Fan et al., 2021; Kalucka et al., 2020)(Figure 5C, see also Table S3 for top 20 genes expressed in each endothelial cell subpopulation cluster). In addition to previously stated marker genes used to identify the pericyte cluster, we have further used *Myl9*, *Notch3* and low expression of *Myh11* for further confirmation. We were able to identify two large vessel EC clusters; arterial ECs (Arterial.EC) enriched for *Hey1*, *Gja4*, *Alpl*, *Sema3g*, *Fbln5, and Stmn2* and venous ECs (Venous.EC) enriched for *Selp*, *Plvap*, *Lrg1*and *Vcam1*. Of interest, in mdxD2-NSG we observed an increase in Venous.EC, compromising about 14% of total EC subpopulations, compared to about 11 and 5% in mdx-NSG and wt-NSG, respectively. We have also noticed a slight decrease in Arterial.EC proportion, at about 10%, compared to 12 and 14% in mdx-NSG and wt-NSG, respectively (Figure 5D).

Recent published scRNA-seq dataset in gastrocnemius muscle from C57BL/6n mouse 8 weeks of age, identified two capillary EC subtypes with one capillary EC population enriched in glycolytic muscle area (Fan et al., 2021). We were similarly able to identify two distinct capillary EC populations, however, with a different gene signature. Capillary.EC.1 expressed *Aqp1*, *Pltp, Txnip*, and *Aqp7* and Capillary.EC.2 expressed *Mme*, *Fbln1* and *Scara5.* Of interest, the proportion of these capillary EC subtypes changes drastically between healthy, dystrophic, and severely dystrophic muscles. Although Capillary.EC.1 contribute the highest proportion of EC in all the muscles, Capillary.EC.2 constitute 2% of total proportion in wt-NSG compared to 4 and 10% in mdx-NSG and mdxD2-NSG, respectively (Figure 5D). We observed a pronounced decrease of both capillary EC subtypes in our dataset from 412 cells in wt-NSG, to 155 and 150 cells in mdx-NSG and mdxD2-NSG, respectively. We subsequently wondered if the decrease in capillary ECs in the dystrophic and severely dystrophic models reflected an increased frequency of stromal cells and macrophages or if there was a true decrease of capillary ECs in the dystrophic and severely dystrophic models. In cross-sections of the gastrocnemius muscle we quantified increased capillary density in mdxD2-NSG compared to both mdx-NSG and wt-NSG, whereas the capillary density of mdx-NSG was not significantly increased compared to wt-NSG (Figure S3A). Our observed result of normal capillary density in the mdx-NSG muscle is similar to previous findings in 3-month-old mdx gastrocnemius muscle (Latroche et al., 2015). To infer differences between the two capillary EC subtypes we performed GO enrichment analysis on DE-Gs in each cluster (Figures S3B and S3C). Capillary.EC.1 top biological processes enriched terms were regulation of angiogenesis and regulation of vasculature development. Unexpectedly, Capillary.EC.2 enriched for ECM organization terms.

In addition, we identified a small EC subtype that expresses an immune profile such as *Ptprc* (CD45), *Cd44*, and *Spp1* in addition to the main EC markers *Pecam1* (CD31) and *Cdh5* (VE-Cadherin) (Figure 5C). Human umbilical cord ECs (HUVECs) have been shown to express CD45 in response to interleukin-1 (IL-1) *in vitro* (Forsyth et al., 1993). Furthermore, in response to TGF-ꞵ it has been shown that mitral valve ECs in the heart express CD45 both *in vitro* and *in vivo* (Bischoff et al., 2016). Therefore, we identified this subtype as an activated subtype of ECs (Activated.EC). It is noteworthy that the Activated.EC subtype constitutes 13% of the total ECs in mdxD2-NSG, 6% in mdx-NSG and only 2% in wt-NSG.

When exploring the DE-Gs upregulated in mdxD2-NSG EC, we found an enrichment for neutrophil degranulation and neutrophil chemotaxis GO terms, with genes enriched such as *Cd63, Fcer1g, Gsn, Ctss* and *Alad,* and enriched GO terms for positive regulation of cell migration, *Calr, Col18a1, Col1a1, Fn1, Plvap* and *Thbs4* (Figures 5E and 5F)*.* Of interest, one of the enriched GO terms in mdxD2-NSG versus mdx-NSG was platelet activation, signaling and aggregation, with genes enriched such as *Cd63*, *Col1a1*, *Fn1*, *Plek*, and *Col3a1* (Figure 5E). Although not shown on the top enriched terms, we also found that the platelet activation, signaling and aggregation enriched GO term, with genes expressed such as *Cd63*, *Clu*, *Col1a1*, *Fcer1g*, *Fn1*, *Plek*, and *Pf4* were upregulated in mdxD2-NSG EC DE-Gs vs. wt-NSG. Taken together, ECs in the severely dystrophic mouse model showed an increased ECM gene upregulation, especially genes involved in collagen formation, regulation of cell adhesion and platelet activation and aggregation. When comparing DE-Gs upregulated in mdx-NSG ECs vs. wt-NSG, enriched terms for ECM organization or degradation was lacking and not evident (not shown). Because of these prominent differences especially in ECM upregulation in the severely dystrophic ECs, which has not been previously shown in pathological muscular dystrophy conditions, we focused our attention into validating these findings.

### Ligand-gene interaction model identifies intercellular communication influencing endothelial cells in dystrophic muscles

**Figure 6:**
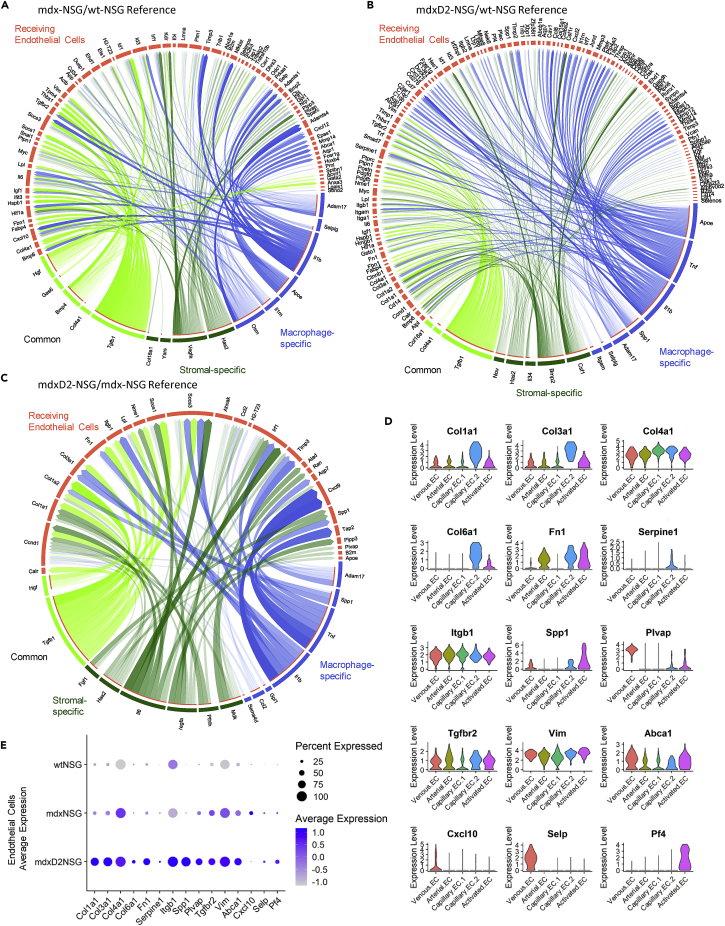
Stromal cells and macrophages released ligands and their regulated target genes on endothelial cells in dystrophic and severely dystrophic environment (A–C) Circos plot of NicheNet analysis showing links of cell-cell interactions between predicted ligands from stromal cells and macrophages with most likely regulated target genes that are differentially expressed on (A) mdx-NSG endothelial cells, wt-NSG as a reference (B) mdxD2-NSG endothelial cells, wt-NSG as a reference, and (C) mdxD2-NSG endothelial cells, wt-NSG as a reference. Degree of transparency is determined by the regulatory potential value of a ligand-target interaction (Blue: macrophage-specific ligands, Green: stromal cell-specific ligands, Lawn green: common ligands released from both stromal cells and macrophages). (D) Violin plots of some genes of interest that were differentially expressed in the dystrophic and severely dystrophic endothelial cells identified by NicheNet analysis. (E) Dot plot of average expression between the mouse models of some genes of interest that were differentially expressed in the dystrophic and severely dystrophic endothelial cells identified by NicheNet analysis.

The skeletal muscle microenvironment and intercellular network interactions is critical in maintaining muscle homeostasis in healthy and diseased conditions. Released ligands from cell populations, stromal cells and macrophages for instance, can govern and drive cellular gene expression changes. Therefore, to address our question of whether the dystrophic microenvironment led to changes and upregulation of ECM gene expression, among others, between healthy, dystrophic and severely dystrophic muscles, we investigated the possible interaction between released ligands from stromal cells and macrophages and their target genes on EC. We carried out NicheNet analysis, a computational tool that uses gene expression data of interacting cells and combines it with existing knowledge to infer ligand-to-target interactions (Browaeys et al., 2020). In our NicheNet analysis, we prioritized ligands released from stromal cells and macrophages driving the differential expression observed in dystrophic and severely dystrophic ECs. Because the prioritization of ligands in NicheNet analysis is not directly related to their expression level, we confirmed the upregulation of the prioritized ligand in diseased conditions. We indeed found some ligands lowly expressed in mdx-NSG and mdxD2-NSG stromal cells and macrophages, therefore when performing further analysis and validation experiments, we only considered target genes affected by upregulated ligands in the diseased conditions (data not shown). The top five predicted ligands upregulated in stromal cells and macrophages most likely influencing gene expression differences in mdx-NSG EC are *Tgfb1*, disintegrin and metalloprotease 17 (*Adam17*), selectin P ligand (*Selplg),* apolipoprotein E (*Apoe)*, and interleukin 1ꞵ (*Il1b*). The top five predicted ligands released in mdxD2-NSG were *Tgfb1*, *Apoe, Tnf*, colony stimulating factor 1 (*Csf1*), *Il1b* (Figures S4A and S4B). We further explored predicted ligands differentially expressed in mdxD2-NSG compared to mdx-NSG stromal cells and macrophages most likely regulating target genes in ECs, and the identified ligands were *Tgfb1*, *Adam17*, bone morphogenetic protein 2 (*Bmp2*), midkine (*Mdk*) and osteopontin (*Spp1*) (Figure S4C). These results come in agreement with extensively studied cytokine and chemokine signaling in dystrophic muscles and are further validated by our scRNA-seq dataset and NicheNet analysis (Kramerova et al., 2019; Paepe and Bleecker, 2013). The heatmap of predicted ligand target genes and their regulatory potential in ECs for mdx-NSG and mdxD2-NSG is shown in Figures S4D–S4F. To confirm target gene differential expression on ECs we plotted the average expression of target genes between diseased and healthy conditions (Figures S4G–S4I). The main intercellular interactions of stromal cell-specific, macrophage-specific, and common ligands and their potential target genes were mapped on circle plots to simplify cell-cell interaction visualization (Figures 6A–6C). We found that the main ligands driving ECM expression in mdxD2-NSG ECs were TGF-ꞵ, ostepontin, TNFα and IL-ꞵ. Although in mdx-NSG EC Hgf and TGFꞵ were the main ligands upregulating ECM expression. Because ECM genes were notably upregulated in mdx-NSG and mdxD2-NSG EC, we simplified their expression visualization, with other genes of interest, in Figures 6D and 6E. Collectively, NicheNet analysis highlighted top predicted ligand interactions released from stromal cells and macrophages with their potential target genes on ECs in dystrophic and severely dystrophic muscles identifying TGF-ꞵ pathway as the main upstream signal driving EC gene expression changes.

### Dystrophic and severely dystrophic endothelial cells have increased ECM deposition

**Figure 7:**
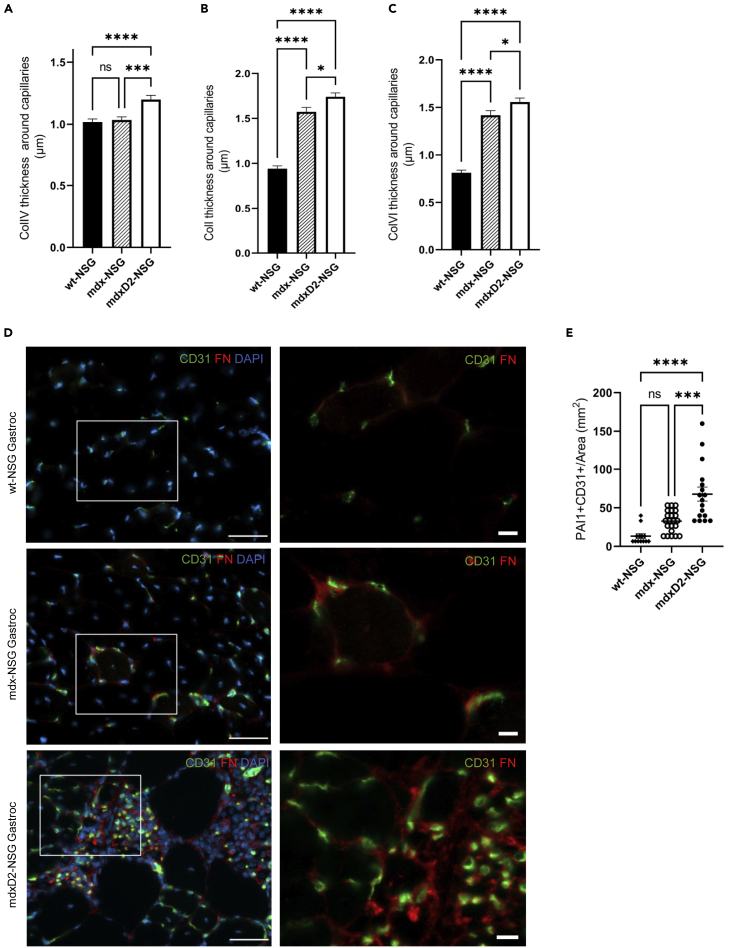
Dystrophic and severely dystrophic endothelial cells have increased ECM deposition (A–C) Quantification of (A) Collagentype IV (COLIV), (B) Collagen I (COLI), (C) Collagen VI (COL VI) deposition around capillary endothelial cells in healthy and DMD muscles. One Way ANOVA with Tukey’s multiple comparison test (Data are represented as mean + SEM ∗p<0.05, ∗∗∗p ≤ 0.0001, ∗∗∗∗p< 0.0001). (D) Immunofluorescence staining of gastrocnemius muscle cross-sections for endothelial cell (CD31, green), and fibronectin (FN, red) and nuclear markers (DAPI, blue). Left column scale bar at 50 μm, right column are magnified images of boxed, scale bar at 10 μm. (E) Quantification of PAI-1 and CD31 colocalization in gastrocnemius muscle cross-sections across the mouse models. One way-ANOVA with Tukey’s multiple comparisons test (Data are represented as mean ± SEM *∗∗∗*p*≤*0.001, ∗∗∗∗p<0.0001).

To validate our findings of increased ECM expression of vascular ECs, especially in severely dystrophic muscles, we examined collagen IV, collagen I, and collagen VI deposition around capillaries in cross sections of wt-NSG, mdx-NSG and mdxD2-NSG muscles (Figures 7A–7C and S5A–S5C). We found significantly higher deposition of collagen IV, collagen I and collagen VI in mdxD2-NSG compared to both mdx-NSG and wt-NSG, whereas higher deposition of collagen I and collagen VI were evident in mdx-NSG compared to wt-NSG (Figures 7A–7C). In addition, one of the major ECM genes that was upregulated in dystrophic and severely dystrophic muscle EC was fibronectin (FN) encoded by *Fn1* (Figure 6E). ECs from murine models are difficult to isolate in large numbers, therefore, to examine the protein expression of fibronectin in the different mouse models we performed western blots (WB) using whole gastrocnemius muscle lysates. We detected significantly increased protein abundance of fibronectin in mdx-NSG and mdxD2-NSG muscles compared to wt-NSG (Figure S6A). When muscle cross-sections were examined, fibronectin had increased deposition around mdx-NSG and mdxD2-NSG ECs compared to wt-NSS (Figure 7D). We further confirmed these findings with Sirius red staining in muscle cross-sections (Figure S6B). Collectively, we confirmed that the upregulation of ECM genes in ECs, regulated mainly by TGF-ꞵ signaling predicted by NicheNet analysis, translates to protein expression in muscle cross-sections of healthy, dystrophic and severely dystrophic muscles.

*Serpine1*, which encodes PAI-1, was one of the genes detected to be upregulated in severely dystrophic ECs in NicheNet analysis (Figure 6B). It was predicted by NicheNet analysis that *Serpine1* is regulated by TGF-β, IL-1ꞵ and TNF signaling (Figure S4E). To further explore this finding, we assessed *Serpine1* expression in ECs and verified its enrichment in mdxD2-NSG EC (Figure 6E). To confirm if our gene expression data corresponds to protein expression, we performed immunostaining of PAI-1 on muscle cross-sections and we found that it colocalizes with some, but not all, ECs (CD31^+^) in mdxD2-NSG (Figures 7E and S6C). Overall, we demonstrate the upregulation of ECM genes by dystrophic and severely dystrophic ECs, and the upregulation of PAI-1 especially in the severely dystrophic ECs indicating EC functional impairment.

## Discussion

Recent advances in scRNA-seq technology have allowed for the evaluation and dissection of skeletal muscle-resident cell populations in homeostatic and regenerating states (Dell’Orso et al., 2019; Giordani et al., 2019; Malecova et al., 2018; Micheli et al., 2020; Oprescu et al., 2020). These studies collectively described a comprehensive scRNA-seq datasets and provided insights into the complexity and diversity of normal muscle-resident populations, and how cell populations change during muscle regeneration. However, limited studies have examined cellular heterogeneity of skeletal muscle at single cell resolution in pathological conditions, for instance in inflammatory muscle diseases such as in DMD mouse models (Camps et al., 2020). Many studies have demonstrated that the diseased environment plays an enormous role in the effectiveness of many therapies (Boisgerault and Mingozzi, 2015; Galli et al., 2021). Therefore, utilizing scRNA-seq, here we focused on evaluating the changes in the cellular composition and microenvironment as disease severity increases in mouse models of DMD. We explored the transcriptional differences and heterogeneity within different muscle-resident populations including MuSCs, stromal cells, macrophages, and EC populations between healthy, dystrophic, and severely dystrophic muscles.

MuSC has been demonstrated to be a heterogeneous population influenced by microenvironment signaling (Motohashi and Asakura, 2014; Tierney and Sacco, 2016). The first scRNA-seq study that focused on MuSC heterogeneity in homeostatic muscle constituted of only 21 FACS isolated MuSCs (Cho and Doles, 2017). Since then, multiple groups examined the temporal dynamics of MuSCs during regeneration where quiescent MuSCs, activated MuSCs (Dell’Orso et al., 2019), progenitors, committed progenitors, mature skeletal muscle (Dell’Orso et al., 2019; Micheli et al., 2020), and immunomyoblasts (Oprescu et al., 2020) have been described. We described a MuSC subpopulation that expressed an immune profile that constitutes about 25% of MuSC in the mdxD2-NSG muscle, similar to described in the regenerating muscle (Oprescu et al., 2020). The functional validation of such population is necessary in the severely dystrophic muscle to understand its implications in the disease context. We further described four MuSC subpopulations that are transcriptionally distinct in healthy, dystrophic and severely dystrophic scRNA-seq integrated dataset, with an increased proportion of committed (*Myod1*+) and differentiated (*Myog*+) MuSC in the dystrophic and severely dystrophic muscle. Furthermore, we were able to identify, in dystrophic and severely dystrophic skeletal muscles, cell populations that have been otherwise described in healthy regenerating muscle after acute injury, such as *Cxcl5*+ stromal cell subpopulation (Micheli et al., 2020; Oprescu et al., 2020). We were able to further describe the dynamic changes of macrophages with the co-existence of both pro-inflammatory, M1-like macrophage, and anti-inflammatory, M2-like macrophage, signatures in the dystrophic and severely dystrophic skeletal muscles. Of interest, we have observed that pericytes differentially express fibrosis markers in mdxD2-NSG and mdx-NSG muscles such as *Postn*, *Col1a1/a2* and *Fn1* compared to wt-NSG(not shown). A similar pro-fibrotic signature of pericytes has been described in heart and brain after ischemic injury (Pham et al., 2021). A common signaling pathway associated with both ischemia injury as well as dystrophic and severely dystrophic environments is exacerbated TGF-ꞵ signaling, which we speculate is resulting in a similar fibrotic signature in the pericytes in dystrophic and severely dystrophic muscles.

EC skeletal muscle-resident population is an understudied cell population although its implications for cell-based therapeutics are critical. In the mdx mouse model, it has been reported that the vascular changes are age-dependent, with impaired angiogenesis, migration, and proliferation of ECs *in vitro* and *in vivo* in older mdx mice (Latroche et al., 2015; Palladino et al., 2013; Podkalicka et al., 2021). To understand the role of the microenvironment in EC transcriptional changes in dystrophic and severely dystrophic muscles, we were able to interrogate intercellular communication with predicted ligands released from stromal cells and macrophages and their targets in dystrophic ECs utilizing NicheNet analysis (Browaeys et al., 2020). Our analysis identified TGF-ꞵ pathway, with expression of *Spp1* and *Tgfb1,* as one of the main upstream signals driving EC changes in both dystrophic and severely dystrophic muscles. Recent work has provided evidence that osteopontin promotes fibrosis and upregulates collagen expression in mdx fibroblasts by enhancing TGF-ꞵ signaling (Kramerova et al., 2019). To the best of our knowledge, we are showing here for the first time that in severely dystrophic environment a subset of capillary ECs upregulates ECM genes including *Col1a1*, *Col3a1*, *Col4a1, Col6a1* and *Fn1*. We further confirm the significant increase of Collagen IV, I, VI and fibronectin deposition around capillaries in severely dystrophic environment compared to both dystrophic and healthy environment. Our findings are in line with reported thickened connective tissue around capillaries of DMD patients (Miike et al., 1987). Nichenet analysis moreover identified PAI-1, encoded by *Serpine1*, as an upregulated gene in ECs. We have also seen a similar upregulation of *Serpine1* in stromal cells and macrophages (data not shown). PAI-1 is a procoagulant and inhibits degradation of clots when they arise (Sillen and Declerck, 2020). We confirmed upregulation of PAI-1 protein expression with immunofluorescence imaging in muscle cross-sections, further indicating EC impairment in severely dystrophic muscles.

In summary, we have used scRNA-seq of gastrocnemius skeletal muscle to investigate differences in the microenvironment and cellular constitution as disease severity increases in mouse models of DMD. We have identified critical changes in transcriptional profiles of MuSC, stromal cells, macrophages and ECs regulated by DMD disease progression. Our scRNA-seq data provides the community with a tool to further explore muscle-resident cell type changes and differences in muscle make up between healthy, dystrophic and severely dystrophic phenotypes. Our work further highlights that future studies will likely need to develop combination therapies targeting both the diseased microenvironment in addition to delivering new cell, genetic or other novel therapeutics to DMD patients.

### Limitations of the study

The confirmation of muscle-resident subpopulations identified in this study will require the use of combination approaches of *in vivo* and *in vitro* studies to validate and determine their role in driving disease progression in dystrophic and severely dystrophic mouse models. Moreover, future studies will require investigating ECs in DMD disease model to evaluate their functional role and their targeting to restore a healthy phenotype. In our hands, ECs were challenging to isolate and culture, even when pooling muscles from multiple mice, therefore ECM deposition was quantified using muscle cross-sections or whole muscle lysates.

## STAR★Methods

### Key resources table

**Table:** 

REAGENT or RESOURCE	SOURCE	IDENTIFIER
**Antibodies**		

Anti-Mouse CD31 (MEC 13.3)	BD Biosciences	Cat# 553370; RRID:AB_394816
Anti-Mouse/Human PAI1 [EPR21850-82]	Abcam	Cat# ab222754
Anti-Mouse/Human Fibronectin antibody	Abcam	Cat# ab2413; RRID:AB_2262874
Anti-Mouse/Human Collagen IV antibody	Abcam	Cat# ab19808; RRID:AB_445160
Anti-Mouse CollagenType VI Antibody	Fitzgerald Industries International	Cat# 70R-CR009X; RRID:AB_1283876
Anti-Mouse CollagenType I	Cedarlane Labs	Cat# CL50151AP-1
Monoclonal Anti-α-Actinin (Sarcomeric) antibody	Sigma-Aldrich	Cat# A7811; RRID:AB_476766
Rabbit IgG Isotype Control (Polyclonal)	R&D	Cat# AB-105-C; RRID:AB_354266
Mouse IgG2a Isotype Control	Biolegend	Cat# 401501
Anti-Rat Alexa fluor 488	Fisher Scientific	Cat# A11006; RRID:AB_2534074
Anti-Rabbit Alexa Fluor 647	Fisher Scientific	Cat# A21245; RRID:AB_2535813
Anti-Rabbit Alexa Fluor 568	Fisher Scientific	Cat# A11011; RRID:AB_143157
Anti-Mouse IgG HRP antibody	Cell Signaling	Cat# 7076P2
Anti-Rabbit HRP antibody	Cell Signaling technology	Cat# 7974S

**Chemicals, peptides, and recombinant proteins**		

Collagenase, Type 2 (Collagenase II)	Worthington-Biochem	Cat# LS004177
Dispase II	Thermo Fisher Scientific	Cat# 17105041
Collagenase D	Sigma-Aldrich	Cat# 11088882001
DMEM/F-12, HEPES medium	Thermo Fisher Scientific	Cat# 11330032
Amphotericin B	Thermo Fisher Scientific	Cat# 15290018
Fetal bovine serum (FBS)	Thermo Fisher Scientific	Cat# 16000044
Bovine Serum Albumin	Sigma Aldrich	Cat# A9418-100G
UltraPure BSA	Thermo Fisher Scientific	Cat# AM2616
Vectashield mounting medium with DAPI	Vector Laboratories	Cat#: H-1200-10
Fast Green FCF	Sigma-Aldrich	Cat#:F7258-25G
Direct Red 80	Sigma-Aldrich	Cat#: 365548-25G
Picric Acid, Saturated	Sigma-Alrdrich	Cat#:5860-16
Xylenes (histological)	Fisher Scientific	Cat#: X3P-1GAL
Ethanol, Absolute (200 Proof)	Fisher Scientific	Cat#: BP2818100
Permount mounting medium	Fisher Scientific	Cat#: SP15-100

**Critical commercial assays**		

Thermo Scientific™ Micro BCA™ Protein Assay Kit	Fisher Scientific	Cat# PI23235
Western Blotting Application Solutions Kit	Cell Signaling Technology	Cat# 12957S
Pierce™ ECL Plus Western Blotting Substrate	Thermo Fisher Scientific	Cat# 32132X3

**Deposited data**		

Raw scRNA-seq data and processed matrices	This paper	GEO: GSE213925
Original western blot images	This paper	Mendeley Data: https://doi.org/10.17632/nptpkvvtjs.1

**Experimental models: Organisms/strains**		

C57Bl/6-NSG	N/A	N/A
mdx-NSG	N/A	N/A
mdxD2-NSG	N/A	N/A

**Software and algorithms**		

Zen 2.6 (blue edition)	Carl Zeiss Microscopy	
Prism 9.1.1	GraphPad	https://www.graphpad.com
CellRanger	10X Genomics	Cell Ranger Installation-Software-Single Cell Gene Expression-Official 10x Genomics Support
Seurat Version 3.1.5	Butler et al. (2018)	Release Version 3.1.5 · satijalab/seurat · GitHub
Harmony	Korsunsky et al. (2019)	GitHub - immunogenomics/harmony: Fast, sensitive and accurate integration of single-cell data with Harmony
NicheNet	Browaeys et al. (2020)	GitHub - saeyslab/nichenetr: NicheNet: predict active ligand-target links between interacting cells
Metascape	Zhou et al. (2019)	Metascape
ImageLab versions 5.1 and 6.1	Bio-Rad Labratories	

### Resource availability

#### Lead contact

Further information and requests for resources and reagents should be directed to the lead contact, April Pyle (apyle@mednet.ucla.edu).

#### Materials availability

This study did not generate new unique reagents. Requests for mouse strains in this study could be shared by lead contact upon request, April Pyle (apyle@mednet.ucla.edu).

### Experimental model and subject details

#### Mice

All animal work was conducted under protocols approved by the UCLA Animal Research Committee (ARC) (ARC-2006-119). Animals used in this study were housed in an immunocompromised core facility. C57BL/6 mice were crossed with NSG mice to generate C57-NSG mice (referred to as wt-NSG). mdx-NSG mice: mdx/C57BL/10 mice were crossed to NSG mice to generate mdx-NSG mice. mdxDBA2 mice were a generous gift from Dr. Melissa Spencer, UCLA, and were crossed to NSG mice to generate mdxD2-NSG mice. Pups were genotyped using TransnetYX to ensure allele mutations. wt-NSG mice were genotyped for *Scid* and *Il2rg* alleles, mdx-NSG were genotyped for *Scid*, *Il2rg* and *mdx* alleles, mdxD2-NSG were genotyped for *Anxa6*, *Ltbp4*, *Scid*, *Il2rg* and *mdx* alleles. All animals used in this study were homogenous for *Il2rg* knockout and *Scid*. All animals used in this study were backcrossed to the original C57Bl/6 and mdxC57Bl/10 backgrounds for at least five generations. Male mice were used in this study.

### Method details

#### Muscle digestion and single-cell suspension preparation

Gastrocnemius muscles were harvested from the right hindlimb of 8 weeksold male wt-NSG, mdx-NSG, and mdxD2-NSG each separately. The muscles were first washed with wash buffer consisting of DMEM/F12, 10% FBS, 0.5% P/S and 0.1% Amphotericin B. The muscles were then finely chopped in at room temperature (RT) in digestion buffer consisting of DMEM/F12, 0.5% P/S, 5% FBS and 500 u/ml Collagenase II. The chopped muscles were then placed in 37°C incubator on a shaker for 20 minutes, pipetting with a serological pipette after 10 minutes for trituration. The digested muscles were then mashed with a 5 ml syringe plunger one for each sample. Digestion was stopped by adding icecold wash buffer to each sample and the tissue slurry was then transferred to 50 ml conical tube. The tubes were then filled with wash buffer and centrifuged at 600 g for 5 minutes. The pellets were resuspended in second digestion buffer consisting of DMEM/F12, 0.5% P/S, 5% FBS, 1.5 u/ml Collagenase D, and 2.4 u/ml Dispase. The tissue was then placed in a 37°C incubator on a shaker for 15 minutes with intermittent trituration. Icecold FACS buffer consisting of 2% FBS, 0.5% P/S in PBS was added to the tubes to stop digestion. The solution was filtered through a 70 um filter and then a 40 um filter. The cell suspension was then centrifuged at 400 g for 5 minutes at 4°C, the pellet was resuspended with FACS buffer and centrifuged at 600 g for 5 minutes at 4°C. Cell number was counted, resuspended in FACS buffer, stained with 1 μg/ml DAPI and kept on ice until sorted.

#### 10Xgenomics library preparation and sequencing

Single-cell suspension prepared as described above was sorted by BD FACSAria sorter for live cells at UCLA Broad Stem Cell Research Center Flow Cytometry Core. Standard gating strategies were applied to exclude debris, doublets and dead cells. The cells were pelleted and washed twice with BSA buffer, consisting of 1 × PBS with 0.04% UltraPure BSA, with 300 g centrifugation between washes for 2 minutes. Cells were then counted and resuspended in a 1200 cells/μl BSA buffer ratio. Chromium™ Single Cell 3′ Library Construction was performed by the Technology Center for Genomics and Bioinformatics (TCGB) at UCLA. The samples were then sequenced using the llumina NovaSeq 6000 S2 (2 × 50).

#### Immunofluorescence staining

Mice gastrocnemius muscles were frozen in isopentane cooled in liquid nitrogen. Frozen muscles were serially sectioned at 10 μm thick cryosections. A Hydrophobic barrier was drawn around sections, then washed with 0.1% Tween in PBS (PBST). The sections were then fixed with 4% PFA for 10 minutes. A permeabilization step, if necessary, followed with 0.3% Triton X-100 in PBS at room temperature for 10 minutes. Sections were then blocked with 0.25% Gelatin, 0.1% Tween, 3% bovine serum albumin (BSA) and 10% goat serum (GS) in distilled water for 60 minutes at room temperature. Sections were then incubated in humidified chambers with either Col-I (1:250), Col-IV (1:250), Col-VI (1:250) or FN (1:250) and CD31 (1:100) primary antibodies overnight at 4°C in 0.25% gelatin, 0.1% Tween, 3% BSA and 1% GS. Sections were next incubated for 60 minutes with fluorophore-conjugated secondary antibodies diluted in PBS and 1% goat serum (1:. DAPI vecatshield mounting media was then used to counterstain nuclei, coverslips were applied, and nail polish was used to seal the coverslips. Images were captured using a Zeiss Axio Observer.Z1 microscope equipped with an AxioCamMR3 camera.

#### Western blot

Muscle lysates and western blotting was carried using Western Blotting Application Solutions Kit from Cell Signaling Technology, following manufacturer protocol. In brief, gastrocnemius muscles were harvested from 11-weeksold male wt-NSG, mdx-NSG and mdxD2-NSG mice (n = 5 of each mouse model) after euthanization, and immediately frozen in liquid nitrogen. Muscle samples were homogenized using Qiagen TissueRuptor in icecold 2X cell lysis buffer supplemented with 1mM PMSF (100 mg of tissue to 1 ml of 2X buffer) and sonicated for 10–15 seconds. Samples were kept on ice at all times. Total proteins were quantified using Pierce Micro BCA protein assay kit. Samples were prepared with 1X SDS sample buffer, diluted from 3X SDS buffer consisting of 187.5 mM Tis-HCl (pH 6.8 at 25°C), 6% (w/v) SDS, 30% glycerol and 0.03% (w/v) bromophenol blue, supplemented with 1/10 volume of 30X DTT reducing agent, consisting of 1.25 M dithiothreitol. Samples were heated to 95–100°C for 5 minutes, and then cooled on ice for 5 minutes. Lysates, with a total protein of 30 μg, were resolved by SDS-PAGE using 1X running buffer carried out using Criterion Vertical Electrophoresis Cell (Bio-Rad) on 4–20% Gels (Bio-Rad) for 70–80 minutes at 120 V (depending on protein size). Gels were then transferred in wet conditions to nitrocellulose membrane using 1X transfer buffer for 120 minutes at 200 mA in cold room. Membranes were then blocked in TBST with 5% nonfat dry milk for 60 minutes at room temperature. Next, the membranes were incubated overnight at 4°C with primary antibodies diluted in 3% BSA in TBST (1:1000). Membranes were then incubated in species appropriate HRP conjugated secondary antibodies (1:2000) for 60 minutes at room temperature and developed with Pierce ECL Western Blotting substrate using ChemiDoc MP Imaging system.

#### Sirius red histological stain

Picrosirius red stain solution was prepared by adding 0.1% direct red 80 and 0.1% fast green FCF to aqueous saturated Picric acid. Slides were then washed in Xylene for 5 minutes (2X), 100% EtOH for 2 minutes (2X), 90% EtOH for 2 minutes (2X), 80% EtOH for 2 minutes (2X), 70% EtOH for 2 minutes (2X), and dH_2_O for 2 minutes (2X). Tissue sections on the slides were then outlined with a pap pen. Picrosirius red solution was applied to the sections and left to incubate for 60 minutes. Slides were then washed in H_2_O (10 dips), 70% EtOH (10 dips), 100% EtOH (2X 10 dips), and Xylene (2X 10 dips). Finally, slides were mounted with Permount mounting medium and imaged.

### Quantification and statistical analysis

#### Computational analysis: Seurat

Read alignment and gene-expression quantification of mouse scRNA-seq data was performed using the CellRanger Count pipeline (version 3.1.0, 10X Genomics). The CellRanger pre-built mouse reference package was used for read alignment (mm10 Ensembl 93). The filtered feature bc matrices output was then used to create Seurat object using Seurat package version 3.1.5. Quality Control (QC), data normalization and scaling, and detection of highly variable features were then carried out for each sample individually, with minimal alterations from the published workflow (Butler et al., 2018). Briefly, quality control was performed by removing cells with fewer genes than 500, 800 and 800 and higher genes than 5000, 6000 and 6000 genes from the analysis in wt-NSG, mdx-NSG and mdxD2-NSG samples, respectively. Moreover, cells with more than 15% of UMIs mapped to mitochondrial genes were removed. Next, the data were normalized, highly variable genes identified and scaled using “SCTransform” with regression of cell cycle scores and percent mitochondrial genes. Next, dimensionality reduction by principal components (PCs) was calculated using “RunPCA” and to estimate the significant number of PCs to be used “ElbowPlot” function was used. Next, the uniform manifold approximation and projection (UMAP) embedding were calculated and visualized using “RunUMAP” and “UMAPPlot”. Unsupervised clustering was carried using “FindNeighbors” and “FindClusters”. Differentially expressed genes were then defined with “FindallMarkers” with Wilcox test, return.thresh set to 0.01, logfc.threshold set to 0.41, and with “min.pct” set to 0.25. Muscle clusters of each replicate from each mouse model were then defined (data not shown). The normalized Seurat objects were then merged using “merge”. We found that for the merged Seurat object normalizing and scaling the data with the following functions “NormalizeData”, “FindVariableFeatures” and “ScaleData”, with regression of cell cycle score, resulted in a more conserved and better clustering of the datasets. Integration was then performed as below.

#### Computational analysis: Harmony integration

The merged Seurat object was used to integrate all cells from the different mouse models using Harmony (Korsunsky et al., 2019). Briefly, after running dimensionality reduction with “RunPCA”, Harmony integration follows with “RunHarmony”. In downstream analysis, Harmony embeddings were used for UMAP embeddings and clustering with “RunUMAP”, “FindNeighbors” and “FindClusters”. Differentially expressed genes between clusters were identified with “FindAllMarkers” using following parameters: test used “Wilcox”, “return.thresh” set to 0.01, minimum expression fold change “logfc.threshold” set to 0.41, and minimum percent of cells expressing a certain gene “min.pct” set to 0.25 and minimum gene difference “min.diff.pct” set to 0.15. Clusters were then identified from the integrated data based on gene expression (Table S2) and visualized. A total of 20,920 cells were integrated after carrying Harmony integration from wt-NSG (4180), mdx-NSG (5723), and mdxD2-NSG (11017) (n = 2/mouse model).

#### Computational analysis: Subset data

To obtain a Seurat object containing only desired cell type of the integrated data, endothelial cells for example, the “subset” function was used. The subset Seurat object goes through Seurat filtering and normalization, and Harmony integration workflows as described above.

#### Computational analysis: NicheNet analysis

NicheNet analysis was performed as published code (nichenetr/seurat_wrapper_circos.md at master · saeyslab/nichenetr · GitHub) (Browaeys et al., 2020). Briefly, the expression data of interacting cells was extracted from Seurat object of the integrated data. Then, the receiver cell population (EC) and sender cell populations (Stromal and Mphage) were defined. The analysis was set as such the gene set of interest are the genes differentially expressed in ECs in dystrophic inflammatory environment compared to healthy state. Therefore, the condition of interest was set to mdx-NSG and mdxD2-NSG (each analyzed separately), and the reference condition was set to wt-NSG. Utilizing a model of already published ligand-target, ligand-receptor network and weighted integrated networks, NicheNet analysis is performed according to the published workflow. The predicted ligand activity inferred active ligand-target links and average expression of inferred ligand activity between conditions are shown in Figure S5. Circos plots were then used to visualize groups the top predicted active ligands according to the strongest expressing cell type.

#### Gene Ontology enrichment analysis

Gene Ontology (GO) enrichment was performed using Metascape (Meta http://metascape.org/gp/index.html#/main/step1scape)(Zhou et al., 2019). In short, all statistically GO/KEGG terms were calculated and used for filtering. Significant terms were then hierarchically clustered into a tree based on statistical similarities. P<0.01.

#### Quantification of immunofluorescence imaging

For capillary density: gastrocnemius muscle cross-sections were stained with CD31 and DAPI. Quantification was performed on n = 3 of each mouse model for all the stainings. A total of 3-20X IF images were taken for 10 gastrocnemius muscle cross-sections per mouse, resulting in about 30-35 images for quantification per mouse. Using ImageJ quantified number of capillaries was then normalized to the total area of images counted. For PAI-1 colocalization with CD31 (5/6-20X IF images/mouse) and COLI, COLIV and COLVI (5/6-40X images/mouse), the Zen 2.6 blue edition was used for quantification. Quantified staining was divided by the area of the quantified images (mm^2^). Quantification was performed in a blinded fashion.

#### Statistical analysis

Prism Graphpad was utilized for statistical analysis (https://www.graphpad.com). The *P* values for IF images quantification such as in COLI, COLIV, COLVI, CD31 and PAI-1, and western blot densitometric analysis were calculated using One Way ANOVA with Tukey’s multiple comparison test. Graphs show mean ± SEM, unless otherwise specified.

## Data Availability

• scRNA-seq raw sequencing reads and processed gene expression matrices have been deposited at NCBI GEO and are publicly available as of the date of publication. The original western blot images are available on Mendeley Data. Accession number and DOI of data are listed in key resources table. • This paper does not report original code. Codes from published packages are documented below and has been adapted to our samples with minimal, to no alterations to the original code. Any additional information required to reanalyze the data reported in this paper is available from the lead contact upon request.

## Supplemental information

Document S1. Figures S1–S6 and Tables S1 and S3

Table S2. Top 20 genes expressed in each muscle cell population cluster, related to main Figure 1
