# Supplementary material for: Single cell sequencing maps skeletal muscle cellular diversity as disease severity increases in dystrophic mouse models
Source: iScience. 2022 Oct 21;25(11):105415. doi: 10.1016/j.isci.2022.105415 (PMC9646951; doi:10.1016/j.isci.2022.105415)
Supplement: Document S1. Figures S1–S6 and Tables S1 and S3 [file mmc1.pdf]

iScience, Volume *25*

Supplemental information

Single cell sequencing maps skeletal muscle cellular diversity as disease severity increases in dystrophic mouse models

Kholoud K. Saleh, Haibin Xi, Corey Switzler, Emily Skuratovsky, Matthew A. Romero, Peggie Chien, Devin Gibbs, Lily Gane, Michael R. Hicks, Melissa J. Spencer, and April D. Pyle

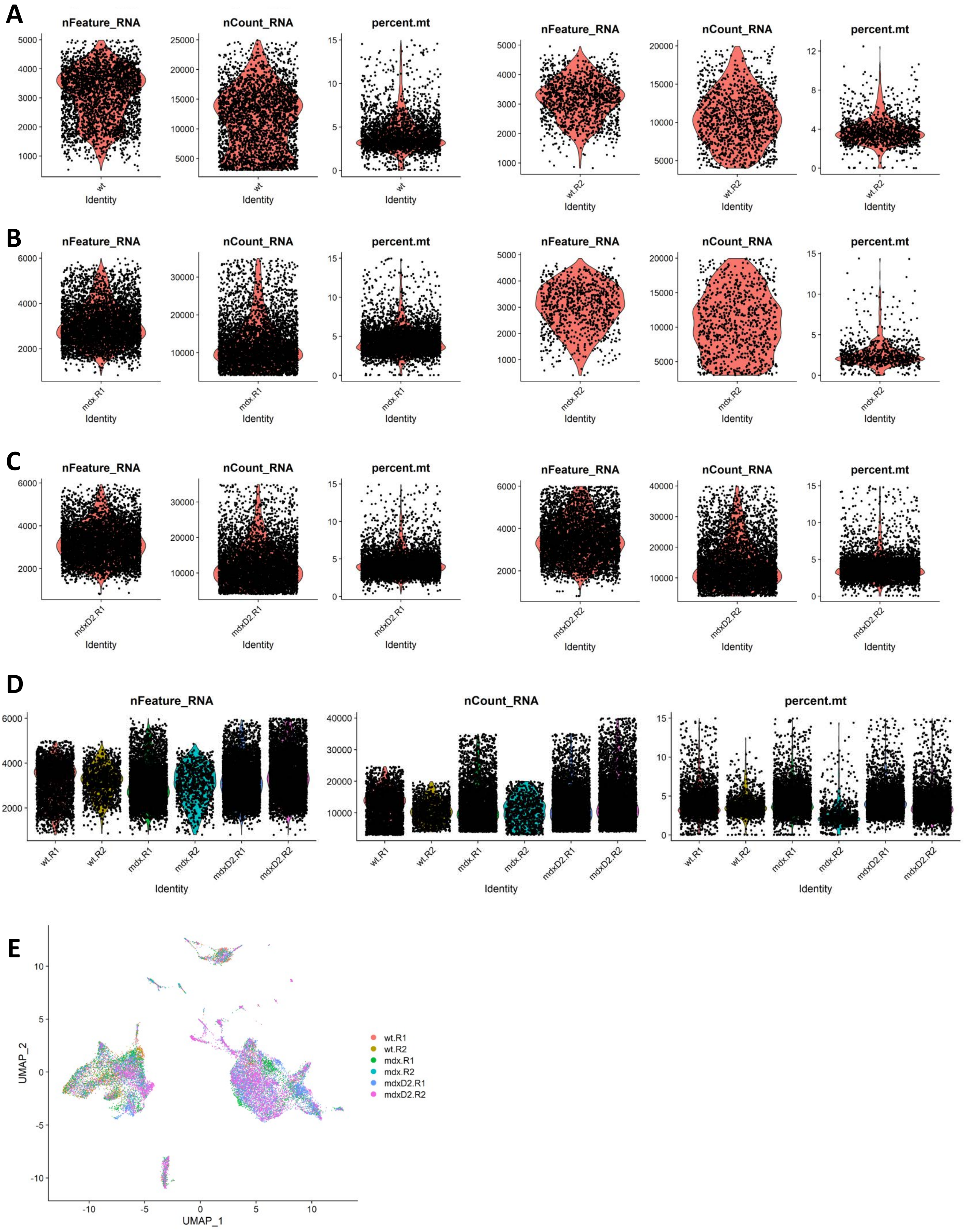
A
nFeature_RNA
nCount_RNA
percent.mt
wt
wt.R2
Identity
B
nFeature_RNA
nCount_RNA
percent.mt
mdx.R1
mdx.R2
Identity
C
nFeature_RNA
nCount_RNA
percent.mt
mdxD2.R1
mdxD2.R2
Identity
D
nFeature_RNA
nCount_RNA
percent.mt
wt.R1
wt.R2
mdx.R1
mdx.R2
mdxD2.R1
mdxD2.R2
Identity
E
UMAP_1
UMAP_2
wt.R1
wt.R2
mdx.R1
mdx.R2
mdxD2.R1
mdxD2.R2

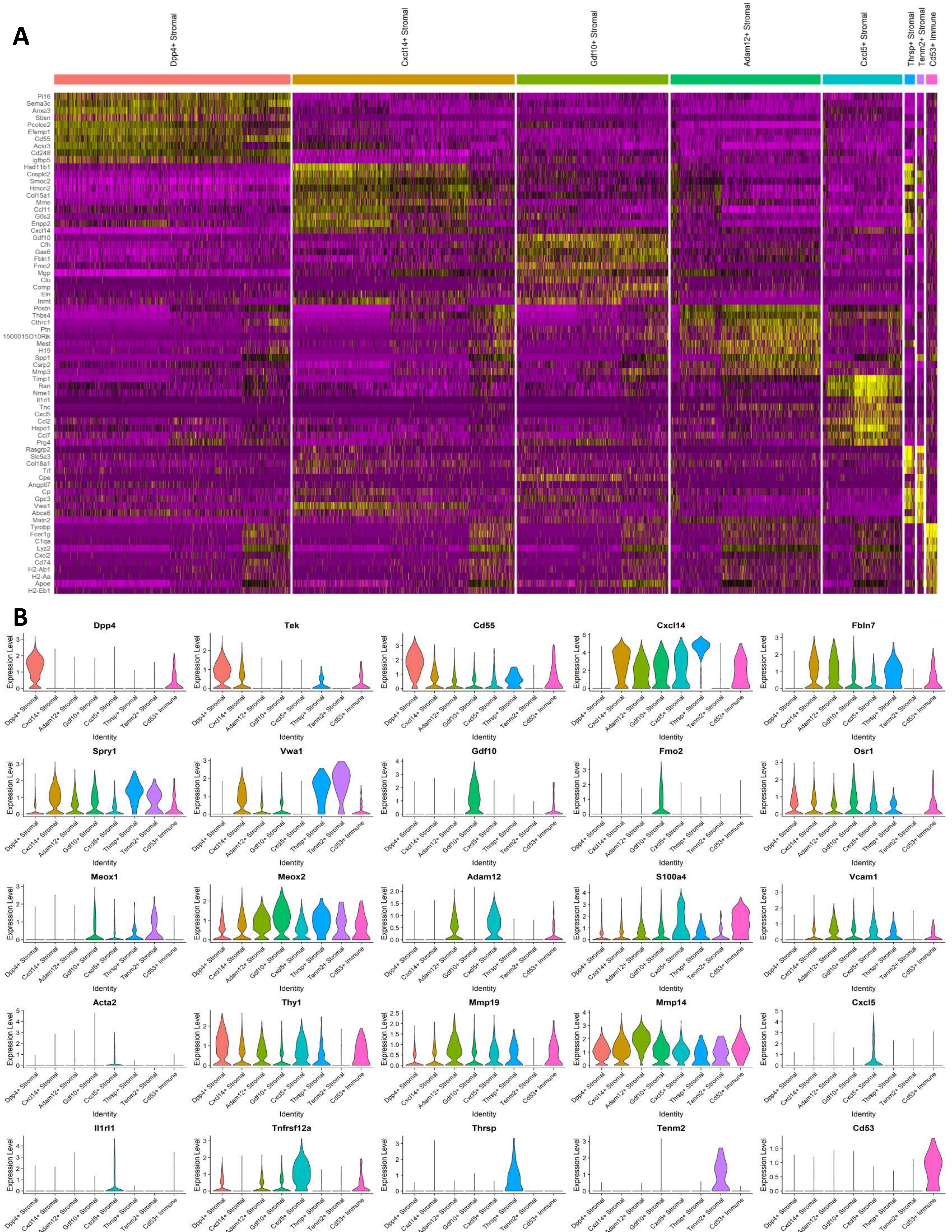

A
Dpp4+ Stromal
Cxcl14+ Stromal
Gdf10+ Stromal
Adam12+ Stromal
Cxcl5+ Stromal
Thrsp+ Stromal
Tenm2+ Stromal
Cd53+ Immune
Pi16
Sema3c
Anxa3
Sbsn
Pcolce2
Efemp1
Cd55
Ackr3
Cd248
Igfbp5
Hsd11b1
Crispld2
Smoc2
Hmcn2
Col15a1
Mme
Ccl11
G0s2
Enpp2
Cxcl14
Gdf10
Cfh
Gas6
Fbln1
Fmo2
Mgp
Clu
Comp
Eln
Inmt
Postn
Thbs4
Cthrc1
Ptn
1500015O10Rik
Mest
H19
Spp1
Csrp2
Mmp3
Timp1
Ran
Nme1
Il1rl1
Tnc
Cxcl5
Ccl2
Hspd1
Ccl7
Prg4
Rasgrp2
Slc5a3
Col18a1
Trf
Cpe
Angptl7
Cp
Gpc3
Vwa1
Abca6
Matn2
Tyrobp
Fcer1g
C1qa
Lyz2
Cxcl2
Cd74
H2-Ab1
H2-Aa
Apoe
H2-Eb1
B
Dpp4
Tek
Cd55
Cxcl14
Fbln7
Spry1
Vwa1
Gdf10
Fmo2
Osr1
Meox1
Meox2
Adam12
S100a4
Vcam1
Acta2
Thy1
Mmp19
Mmp14
Cxcl5
Il1rl1
Tnfrsf12a
Thrsp
Tenm2
Cd53
Expression Level
Identity
Dpp4+ Stromal
Cxcl14+ Stromal
Adam12+ Stromal
Gdf10+ Stromal
Cxcl5+ Stromal
Thrsp+ Stromal
Tenm2+ Stromal
Cd53+ Immune

A

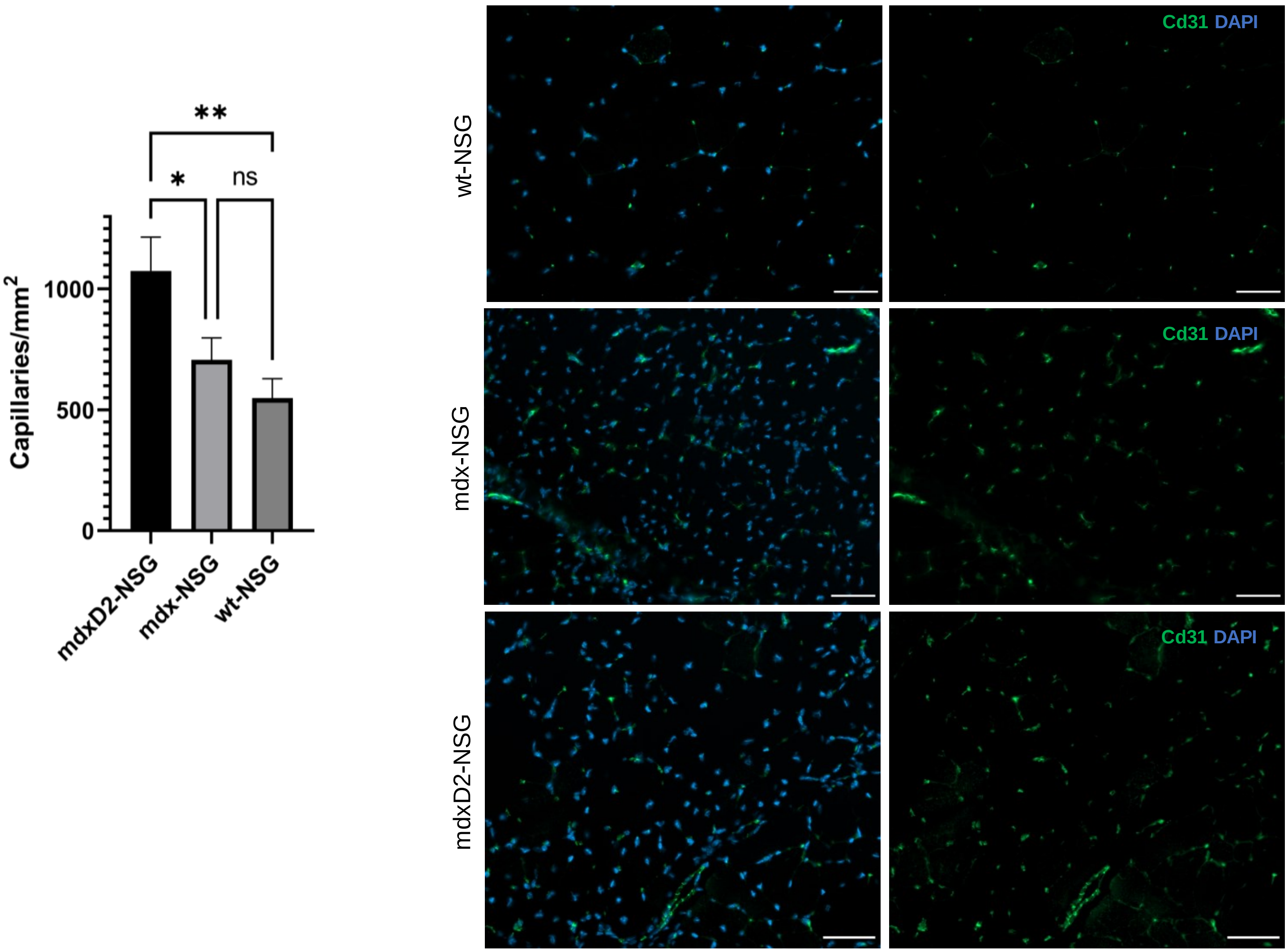

**
*
ns
Capillaries/mm²
1000
500
0
mdxD2-NSG
mdx-NSG
wt-NSG
wt-NSG
mdx-NSG
mdxD2-NSG
Cd31 DAPI
Cd31 DAPI
Cd31 DAPI

B

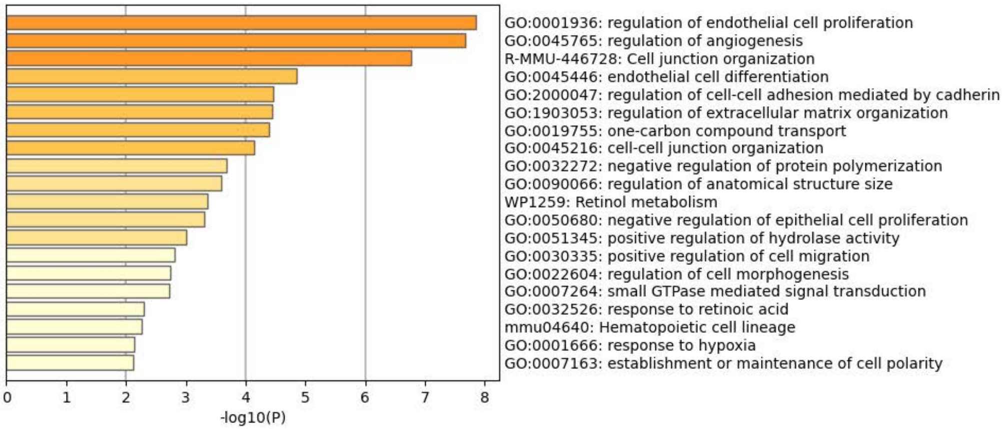

GO:0001936: regulation of endothelial cell proliferation
GO:0045765: regulation of angiogenesis
R-MMU-446728: Cell junction organization
GO:0045446: endothelial cell differentiation
GO:2000047: regulation of cell-cell adhesion mediated by cadherin
GO:1903053: regulation of extracellular matrix organization
GO:0019755: one-carbon compound transport
GO:0045216: cell-cell junction organization
GO:0032272: negative regulation of protein polymerization
GO:0090066: regulation of anatomical structure size
WP1259: Retinol metabolism
GO:0050680: negative regulation of epithelial cell proliferation
GO:0051345: positive regulation of hydrolase activity
GO:0030335: positive regulation of cell migration
GO:0022604: regulation of cell morphogenesis
GO:0007264: small GTPase mediated signal transduction
GO:0032526: response to retinoic acid
mmu04640: Hematopoietic cell lineage
GO:0001666: response to hypoxia
GO:0007163: establishment or maintenance of cell polarity
0 1 2 3 4 5 6 7 8
-log10(P)

C

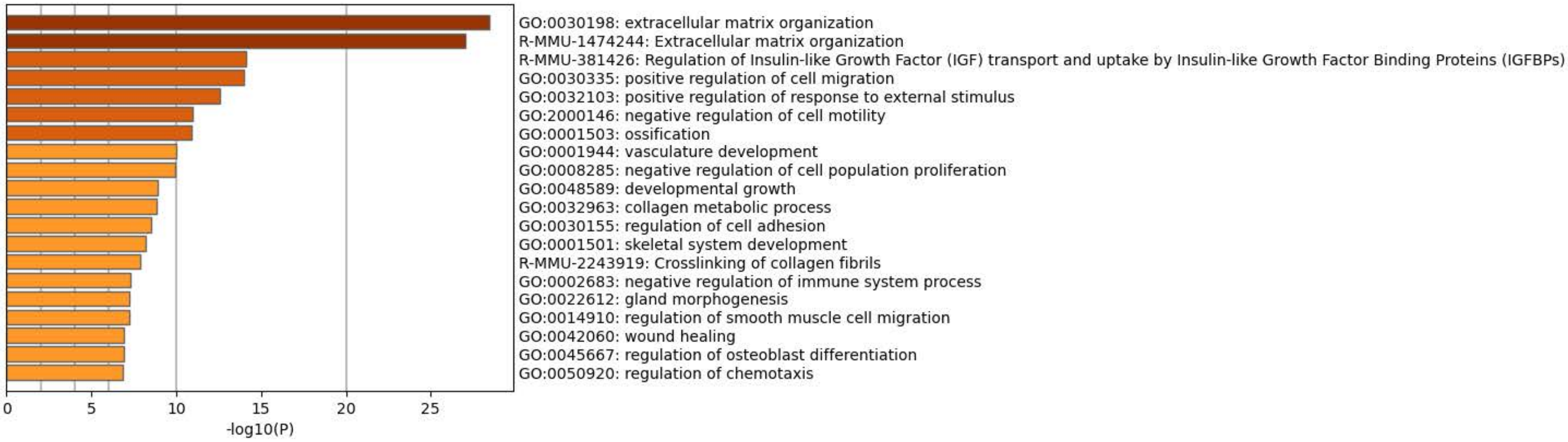

GO:0030198: extracellular matrix organization
R-MMU-1474244: Extracellular matrix organization
R-MMU-381426: Regulation of Insulin-like Growth Factor (IGF) transport and uptake by Insulin-like Growth Factor Binding Proteins (IGFBPs)
GO:0030335: positive regulation of cell migration
GO:0032103: positive regulation of response to external stimulus
GO:2000146: negative regulation of cell motility
GO:0001503: ossification
GO:0001944: vasculature development
GO:0008285: negative regulation of cell population proliferation
GO:0048589: developmental growth
GO:0032963: collagen metabolic process
GO:0030155: regulation of cell adhesion
GO:0001501: skeletal system development
R-MMU-2243919: Crosslinking of collagen fibrils
GO:0002683: negative regulation of immune system process
GO:0022612: gland morphogenesis
GO:0014910: regulation of smooth muscle cell migration
GO:0042060: wound healing
GO:0045667: regulation of osteoblast differentiation
GO:0050920: regulation of chemotaxis
0 5 10 15 20 25
-log10(P)

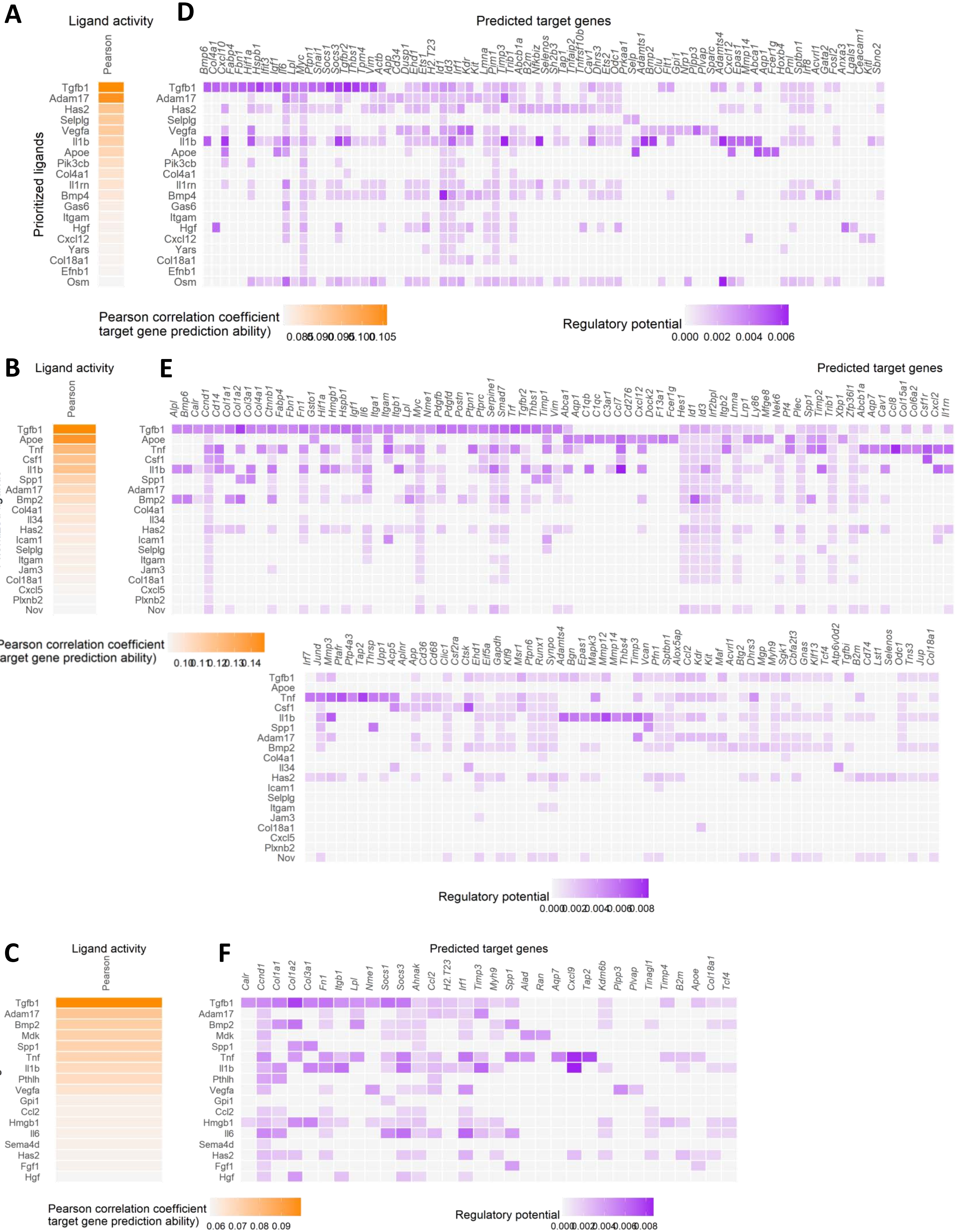

A
Ligand activity
Pearson
Prioritized ligands
Tgfb1
Adam17
Has2
Selplg
Vegfa
Il1b
Apoe
Pik3cb
Col4a1
Il1rn
Bmp4
Gas6
Itgam
Hgf
Cxcl12
Yars
Col18a1
Efnb1
Osm
Pearson correlation coefficient
target gene prediction ability)
D
Predicted target genes
Regulatory potential
0.000 0.002 0.004 0.006
B
Ligand activity
Pearson
Prioritized ligands
Tgfb1
Apoe
Tnf
Csf1
Il1b
Spp1
Adam17
Bmp2
Col4a1
Il34
Has2
Icam1
Selplg
Itgam
Jam3
Col18a1
Cxcl5
Plxnb2
Nov
Pearson correlation coefficient
target gene prediction ability)
E
Predicted target genes
Regulatory potential
C
Ligand activity
Pearson
Prioritized ligands
Tgfb1
Adam17
Bmp2
Mdk
Spp1
Tnf
Il1b
Pthlh
Vegfa
Gpi1
Ccl2
Hmgb1
Il6
Sema4d
Has2
Fgf1
Hgf
Pearson correlation coefficient
target gene prediction ability)
0.06 0.07 0.08 0.09
F
Predicted target genes
Calr
Ccnd1
Col1a1
Col1a2
Col3a1
Fn1
Itgb1
Lpl
Nme1
Socs1
Socs3
Ahnak
Ccl2
H2.T23
Irf1
Timp3
Myh9
Spp1
Alad
Ran
Aqp7
Cxcl9
Tap2
Kdm6b
Plpp3
Plvap
Tinagl1
Timp4
B2m
Apoe
Col18a1
Tcf4
Regulatory potential

G

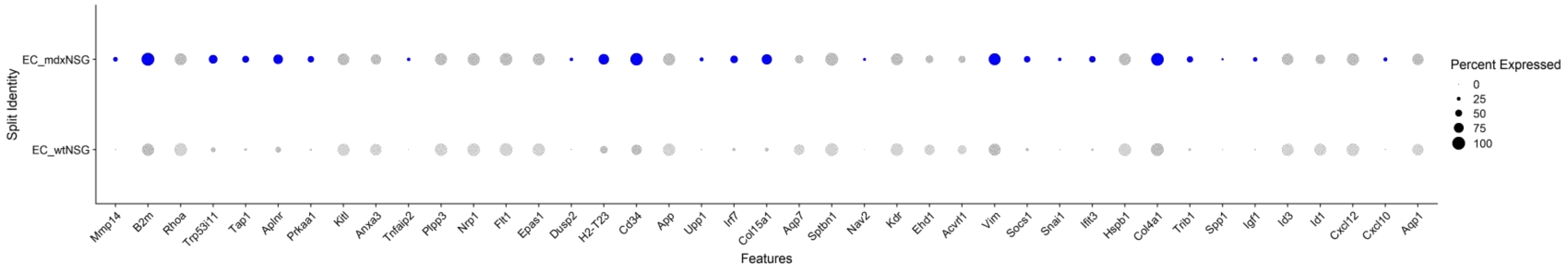
Split Identity
EC_mdxNSG
EC_wtNSG
Mmp14
B2m
Rhoa
Trp53i11
Tap1
Aplnr
Prkaa1
Kitl
Anxa3
Tnfaip2
Plpp3
Nrp1
Flt1
Epas1
Dusp2
H2-T23
Cd34
App
Upp1
Irf7
Col15a1
Aqp7
Sptbn1
Nav2
Kdr
Ehd1
Acvrl1
Vim
Socs1
Snai1
Ifit3
Hspb1
Col4a1
Trib1
Spp1
Igf1
Id3
Id1
Cxcl12
Cxcl10
Aqp1
Features
Percent Expressed
0
25
50
75
100

H

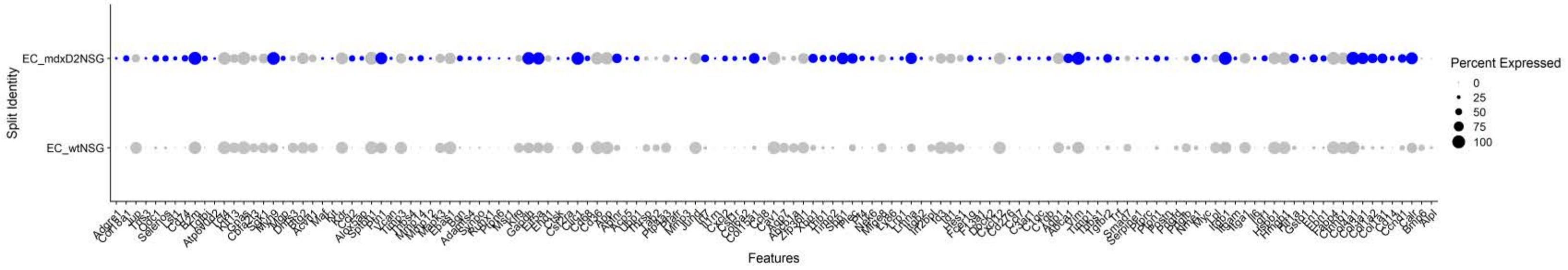
Split Identity
EC_mdxD2NSG
EC_wtNSG
Features
Percent Expressed
0
25
50
75
100

I

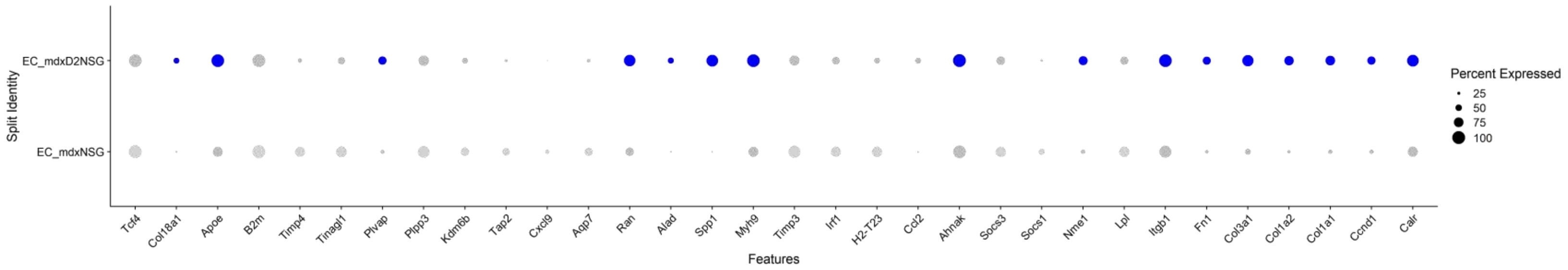
Split Identity
EC_mdxD2NSG
EC_mdxNSG
Tcf4
Col18a1
Apoe
B2m
Timp4
Tinagl1
Plvap
Plpp3
Kdm6b
Tap2
Cxcl9
Aqp7
Ran
Alad
Spp1
Myh9
Timp3
Irf1
H2-T23
Ccl2
Ahnak
Socs3
Socs1
Nme1
Lpl
Itgb1
Fn1
Col3a1
Col1a2
Col1a1
Ccnd1
Calr
Features
Percent Expressed
25
50
75
100

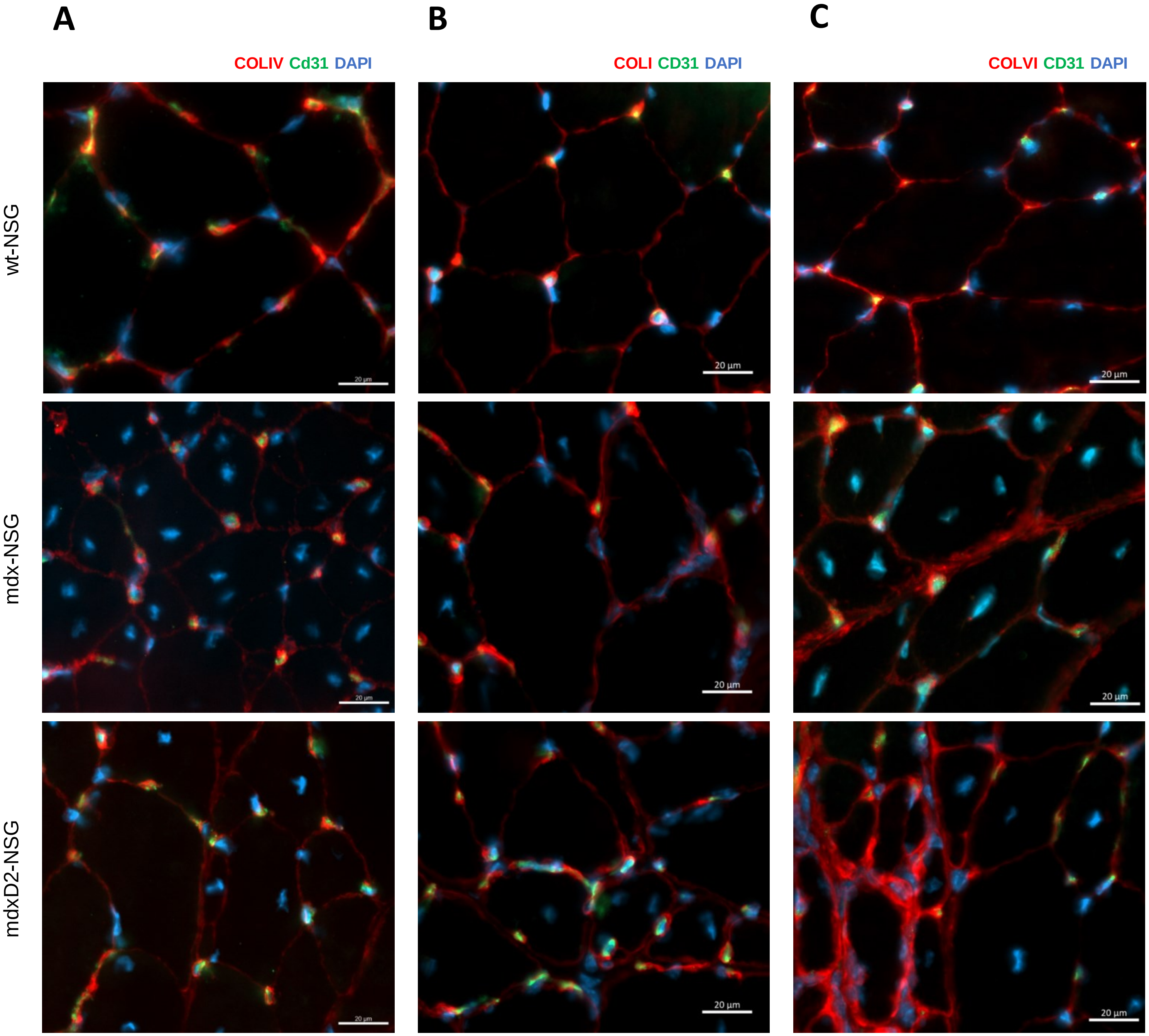
A
B
C
COLIV Cd31 DAPI
COLI CD31 DAPI
COLVI CD31 DAPI
wt-NSG
mdx-NSG
mdxD2-NSG
20 µm

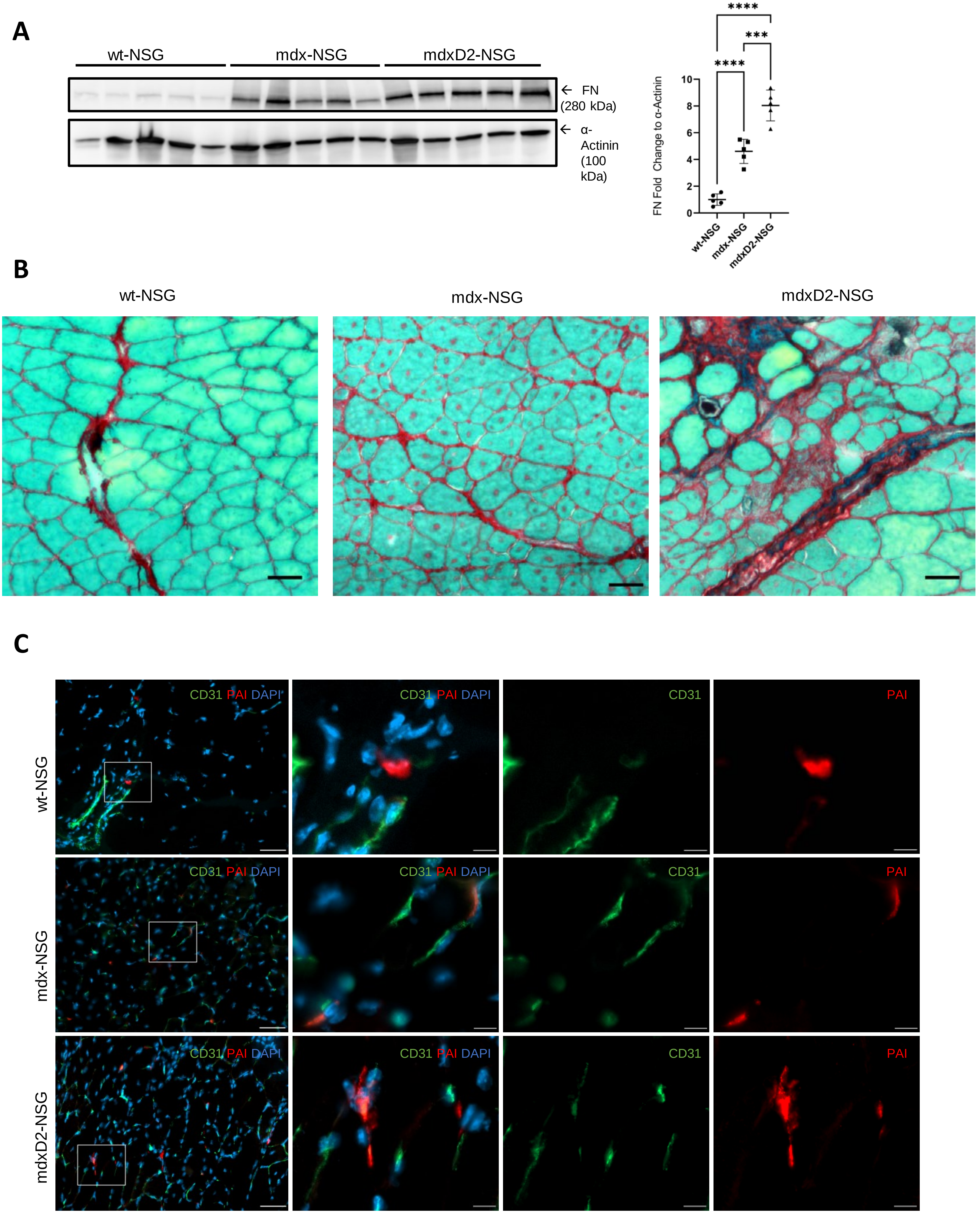

A
wt-NSG
mdx-NSG
mdxD2-NSG
FN
(280 kDa)
α-Actinin
(100 kDa)
****
***
****
FN Fold Change to α-Actinin
10
8
6
4
2
0
wt-NSG
mdx-NSG
mdxD2-NSG
B
wt-NSG
mdx-NSG
mdxD2-NSG
C
CD31 PAI DAPI
CD31
PAI
wt-NSG
mdx-NSG
mdxD2-NSG

SUPPLEMENTAL INFORMATION

Supplemental Figures legends

Figure S1 Related to Figure 1: Quality control for scRNA-seq

Violin plots of analyzed samples showing the distribution of the number of genes (nFeature_RNA), number of RNA counts (nCount_RNA) and percent mitochondrial RNA (percent.mt) in (A) replicate 1 and 2 of wt-NSG gastrocnemius muscles. (B) replicate 1 and 2 of mdx-NSG gastrocnemius muscles. (C) Replicate 1 and 2 of gastrocnemius mdxD2-NSG muscles. (D) The integration of all sample replicates. (E) UMAP embedding of all muscles integrated grouped by replicates.

Figure S2 related to Figure 3: Gene expression profile of stromal cell subtypes.

(A) Heatmap of top 10 genes expressed in each stromal cell subtype.

(B) Violin plot of an expanded panel gene expression used to identify stromal cells subtypes.

Figure S3 related to Figure 5: Capillary endothelial cell differences

(A) Left: capillary Density of 8 weeks gastrocnemius muscles in each mouse model. Right: IF images of representative gastrocnemius muscle cross-sections of the mouse models used for quantification, only capillaries were counted per cross-section. Scale bar on IF images are at 20 µm. Quantification was performed using ImageJ on at least 30 images for each mouse, n=3 per mouse model. One Way-ANOVA with Tukey's multiple comparison test, mean+SD, *P<0.05, **P<0.01

(B) GO enriched terms of DEGs in Capillary EC.1.

(C) GO enriched terms of DEGS in Capillary EC.2.

Figure S4 related to Figure 6: Outcome of NicheNet's ligand activity prediction released from stromal cells and macrophages in dystrophic muscle and their target gene expression regulated on EC.

Outcome of NicheNet's ligand activity prediction of stromal cells and macrophages in (A) mdx-NSG (wt-NSG as reference). (B) mdxD2-NSG (wt-NSG as reference) and (C) mdxD2-NSG (mdx-NSG as reference). Pearson correlation coefficient was used between prior regulatory potential scores and endothelial cell gene set assignments. Pearson correlation indicates the ability of each ligand to predict the endothelial cell (EC) target gene, therefore better predictive ligands are ranked higher.

NicheNet's ligand-target matrix denoting the regulatory potential between predicted released ligands and target gene from EC in (D) mdx-NSG ECs and (E) mdxD2-NSG ECs (F) mdxD2-NSG ECs (mdx-NSG as reference).

(G) Dot plot showing the average expression of NicheNet's predicted target genes on ECs in wt-NSG and mdx-NSG. (H) Dot plot showing the average expression of NicheNet's predicted target genes on ECs in wt-NSG and mdxD2-NSG. (I) Dot plot showing the average expression of NicheNet's predicted target genes on ECs in mdxD2- NSG and mdx-NSG

Figure S5 related to Figure 7: ECM protein staining in wt-NSG, mdx-NSG and mdxD2-NSG of gastrocnemius muscle cross sections

Immunofluorescence staining of gastrocnemius muscle cross-sections for vasculature (CD31, green), and nuclear marker (DAPI, blue) and column (A) collagen type IV (COLIV, red), (B) collagen type I (COLI, red), (C) Collagen type VI (COLVI, red) (Scale bar at 20 µm).

Figure S6 related to figure 7: Validation of fibronectin and plasminogen activator inhibitor-1 upregulation in severely dystrophic gastrocnemius muscle

(A) Fibronectin (FN) expression analysis by western blotting in whole muscle lysates of healthy, dystrophic and severely dystrophic muscles (graph show individual data points of fold change to α-Actinin, One way ANOVA with Tukey's multiple comparisons, mean±SD. ***$P \leq 0.001$, ****$P < 0.001$)

(B) Sirius red stained gastrocnemius skeletal muscle cross-sections of healthy, dystrophic and severely dystrophic mouse models (Scale bar at 50 µm).

(C) Immunofluorescence staining for plasminogen activator inhibitor-1 (PAI-1) and CD31 colocalization of gastrocnemius skeletal muscles across the mouse models.

Table S1 related to Figure 1: Cell counts per mouse model

Sample	Cell subpopulation	Cell Number	Proportion
wtNSG	MuSC	97	2.320574
wtNSG	MC	62	1.483254
wtNSG	EC	519	12.41627
wtNSG	Stromal	2604	62.29665
wtNSG	Peri	78	1.866029
wtNSG	Teno	130	3.110048
wtNSG	Schwann	10	0.239234
wtNSG	Mphage	639	15.28708
wtNSG	Neutro	30	0.717703
wtNSG	Mast	11	0.263158
mdxNSG	MuSC	123	2.149222
mdxNSG	MC	57	0.995981
mdxNSG	EC	222	3.879084
mdxNSG	Stromal	2446	42.73982
mdxNSG	Peri	33	0.576621
mdxNSG	Teno	216	3.774244
mdxNSG	Schwann	5	0.087367
mdxNSG	Mphage	2560	44.73178
mdxNSG	Neutro	38	0.663987
mdxNSG	Mast	10	0.174734
mdxNSG	DC	13	0.227154
mdxD2NSG	MuSC	49	0.444767
mdxD2NSG	MC	16	0.14523
mdxD2NSG	EC	249	2.260143
mdxD2NSG	Stromal	2738	24.8525
mdxD2NSG	Peri	49	0.444767
mdxD2NSG	Teno	330	2.995371
mdxD2NSG	Schwann	10	0.090769
mdxD2NSG	Mphage	7426	67.40492
mdxD2NSG	Neutro	64	0.58092
mdxD2NSG	Mast	6	0.054461
mdxD2NSG	DC	80	0.72615

Table S3 related to Figure 5: Top 20 genes expressed in each endothelial cell subpopulation cluster

p_val	p_val_adj	avg_logFC	pct.1	pct.2	pct.diff	cluster	gene
1.70E-98	3.37E-94	1.062243	0.99	0.733	0.257	Capillary.EC.1	*Gpihbp1*
9.88E-82	1.95E-77	1.131859	0.947	0.574	0.373	Capillary.EC.1	*Rgcc*
2.24E-68	4.43E-64	0.865167	0.962	0.727	0.235	Capillary.EC.1	*Kdr*
2.98E-68	5.90E-64	0.865231	0.96	0.746	0.214	Capillary.EC.1	*Tspan13*
1.72E-66	3.39E-62	0.863168	0.916	0.61	0.306	Capillary.EC.1	*Cd300lg*
1.56E-64	3.08E-60	0.626818	0.999	0.824	0.175	Capillary.EC.1	*Cdh5*
3.70E-64	7.31E-60	1.065456	0.948	0.636	0.312	Capillary.EC.1	*Aqp1*
4.18E-58	8.27E-54	0.758685	0.94	0.778	0.162	Capillary.EC.1	*Cd200*
7.53E-56	1.49E-51	0.891523	0.938	0.763	0.175	Capillary.EC.1	*Tcf15*
4.85E-55	9.60E-51	1.204337	0.789	0.445	0.344	Capillary.EC.1	*Timp4*
1.86E-54	3.68E-50	0.80661	0.913	0.737	0.176	Capillary.EC.1	*Cav2*
6.22E-53	1.23E-48	0.831158	0.808	0.487	0.321	Capillary.EC.1	*Clic5*
7.30E-53	1.44E-48	0.66859	0.996	0.841	0.155	Capillary.EC.1	*Ly6c1*
1.03E-52	2.04E-48	0.682497	0.872	0.659	0.213	Capillary.EC.1	*Ctnnbip1*
2.54E-51	5.02E-47	0.738827	0.914	0.646	0.268	Capillary.EC.1	*C1qtnf9*
1.17E-49	2.31E-45	0.718219	0.841	0.57	0.271	Capillary.EC.1	*Jup*
1.31E-49	2.58E-45	0.779908	0.866	0.646	0.22	Capillary.EC.1	*Lims2*
1.22E-48	2.41E-44	0.685654	0.942	0.767	0.175	Capillary.EC.1	*Afdn*
6.95E-47	1.38E-42	0.811243	0.897	0.676	0.221	Capillary.EC.1	*Tcim*
1.07E-44	2.11E-40	0.966124	0.58	0.208	0.372	Capillary.EC.1	*Car4*
1.62E-44	3.20E-40	0.547063	0.944	0.75	0.194	Capillary.EC.1	*Emcn*
1.29E-90	2.56E-86	1.137749	0.59	0.013	0.577	Capillary.EC.2	*Scara5*
4.09E-77	8.10E-73	0.536022	0.41	0.005	0.405	Capillary.EC.2	*Mme*
6.00E-75	1.19E-70	1.300116	0.692	0.032	0.66	Capillary.EC.2	*Abi3bp*
1.34E-69	2.65E-65	0.750619	0.538	0.016	0.522	Capillary.EC.2	*Svep1*
4.34E-66	8.59E-62	0.585115	0.538	0.018	0.52	Capillary.EC.2	*Zfhx4*
2.94E-64	5.81E-60	0.487362	0.385	0.006	0.379	Capillary.EC.2	*Sox9*
3.96E-64	7.84E-60	0.964631	0.538	0.02	0.518	Capillary.EC.2	*Fbln1*
3.81E-62	7.54E-58	0.960262	0.513	0.018	0.495	Capillary.EC.2	*Gfpt2*
2.43E-58	4.80E-54	1.075102	0.513	0.021	0.492	Capillary.EC.2	*Dpep1*
1.09E-55	2.15E-51	1.194491	0.667	0.045	0.622	Capillary.EC.2	*Fndc1*
1.27E-55	2.50E-51	0.655964	0.487	0.019	0.468	Capillary.EC.2	*Podn*
2.30E-55	4.55E-51	1.001715	0.641	0.041	0.6	Capillary.EC.2	*Pdgfra*
7.12E-51	1.41E-46	1.281911	0.487	0.023	0.464	Capillary.EC.2	*Angptl1*
2.36E-49	4.68E-45	0.644649	0.436	0.017	0.419	Capillary.EC.2	*Arhgap20*
6.31E-49	1.25E-44	1.099917	0.615	0.044	0.571	Capillary.EC.2	*Loxl1*
6.74E-49	1.33E-44	1.238539	0.59	0.04	0.55	Capillary.EC.2	*Htra3*
2.29E-48	4.54E-44	0.757599	0.538	0.031	0.507	Capillary.EC.2	*Scn7a*
7.91E-48	1.57E-43	0.677453	0.487	0.024	0.463	Capillary.EC.2	*Lpar1*
4.74E-46	9.38E-42	0.564926	0.564	0.036	0.528	Capillary.EC.2	*Egfr*
6.07E-46	1.20E-41	0.535627	0.41	0.016	0.394	Capillary.EC.2	*Scara3*
2.82E-152	5.58E-148	0.681372	0.768	0.011	0.757	Activated.EC	*Arhgap30*

1.20E-148	2.37E-144	1.227475	0.826	0.019	0.807	Activated.EC	*Fcgr2b*
1.17E-144	2.31E-140	1.308057	0.884	0.03	0.854	Activated.EC	*Fyb*
6.71E-141	1.33E-136	1.156838	0.725	0.012	0.713	Activated.EC	*Ms4a6b*
1.32E-139	2.61E-135	1.153249	0.841	0.026	0.815	Activated.EC	*Adgre1*
5.90E-139	1.17E-134	1.83153	0.899	0.037	0.862	Activated.EC	*Cybb*
3.69E-138	7.30E-134	0.998384	0.855	0.028	0.827	Activated.EC	*Ncf2*
1.85E-137	3.67E-133	1.321298	0.826	0.026	0.8	Activated.EC	*C3ar1*
3.35E-137	6.64E-133	1.099285	0.855	0.03	0.825	Activated.EC	*Spi1*
2.94E-136	5.81E-132	0.711061	0.609	0.004	0.605	Activated.EC	*Tnfaip8l2*
4.74E-136	9.38E-132	1.157516	0.812	0.024	0.788	Activated.EC	*Aif1*
1.54E-135	3.05E-131	0.810659	0.768	0.019	0.749	Activated.EC	*Clec4a1*
3.62E-135	7.17E-131	1.588221	0.855	0.031	0.824	Activated.EC	*Ms4a6c*
7.76E-134	1.54E-129	1.39379	0.913	0.041	0.872	Activated.EC	*Ptprc*
1.04E-133	2.05E-129	0.739551	0.667	0.009	0.658	Activated.EC	*Dock2*
1.21E-132	2.40E-128	1.230584	0.855	0.031	0.824	Activated.EC	*Ly86*
1.36E-130	2.70E-126	0.957598	0.768	0.021	0.747	Activated.EC	*Csf2ra*
3.29E-130	6.51E-126	0.731448	0.667	0.01	0.657	Activated.EC	*Epsti1*
1.72E-129	3.39E-125	0.966317	0.696	0.014	0.682	Activated.EC	*Fcgr1*
1.01E-128	1.99E-124	1.990227	0.942	0.055	0.887	Activated.EC	*Laptm5*
4.66E-87	9.22E-83	1.302079	0.664	0.06	0.604	Arterial.EC	*Alpl*
1.71E-86	3.38E-82	1.458309	0.744	0.093	0.651	Arterial.EC	*Sema3g*
1.71E-84	3.38E-80	1.770119	0.8	0.127	0.673	Arterial.EC	*Fbln5*
2.46E-80	4.87E-76	1.729555	0.88	0.177	0.703	Arterial.EC	*Stmn2*
5.95E-80	1.18E-75	1.017539	0.456	0.018	0.438	Arterial.EC	*Gja5*
2.67E-79	5.28E-75	1.148044	0.624	0.06	0.564	Arterial.EC	*Col8a1*
1.80E-67	3.56E-63	0.789036	0.528	0.045	0.483	Arterial.EC	*Eps8l2*
4.49E-61	8.89E-57	0.706346	0.472	0.037	0.435	Arterial.EC	*Crispld1*
1.30E-60	2.58E-56	0.782563	0.488	0.043	0.445	Arterial.EC	*St8sia6*
1.63E-60	3.22E-56	1.355213	0.784	0.166	0.618	Arterial.EC	*Gja4*
1.46E-58	2.90E-54	1.206113	0.704	0.13	0.574	Arterial.EC	*Hey1*
2.07E-58	4.09E-54	0.464141	0.264	0.002	0.262	Arterial.EC	*Nebl*
1.38E-50	2.74E-46	1.280464	0.6	0.104	0.496	Arterial.EC	*Edn1*
3.49E-42	6.90E-38	0.933705	0.512	0.09	0.422	Arterial.EC	*Vegfc*
1.73E-37	3.42E-33	0.476869	0.288	0.022	0.266	Arterial.EC	*Alox12*
3.96E-35	7.83E-31	0.67643	0.44	0.077	0.363	Arterial.EC	*Atp2a3*
1.46E-33	2.88E-29	0.760157	0.432	0.077	0.355	Arterial.EC	*Tgfb2*
1.46E-32	2.89E-28	0.831464	0.464	0.099	0.365	Arterial.EC	*Palld*
3.06E-32	6.06E-28	0.551942	0.392	0.061	0.331	Arterial.EC	*Car7*
5.44E-31	1.08E-26	1.000036	0.76	0.351	0.409	Arterial.EC	*Pdcd4*
7.03E-138	1.39E-133	2.182839	0.782	0.025	0.757	Venous.EC	*Selp*
1.62E-104	3.21E-100	1.273694	0.828	0.062	0.766	Venous.EC	*Chp2*
7.25E-81	1.43E-76	1.220817	0.69	0.056	0.634	Venous.EC	*Slco2a1*
6.41E-77	1.27E-72	2.120923	1	0.217	0.783	Venous.EC	*Plvap*
4.35E-75	8.60E-71	2.06285	0.897	0.146	0.751	Venous.EC	*Lrg1*
3.61E-74	7.15E-70	0.845658	0.575	0.039	0.536	Venous.EC	*Ctnnal1*
3.88E-64	7.67E-60	1.898819	0.954	0.239	0.715	Venous.EC	*Vwf*

6.09E-63	1.20E-58	1.171017	0.862	0.156	0.706	Venous.EC	*Ptgs1*
7.75E-61	1.53E-56	1.206662	0.356	0.011	0.345	Venous.EC	*Ackr1*
1.25E-56	2.48E-52	0.832853	0.644	0.076	0.568	Venous.EC	*Cysltr1*
4.21E-56	8.33E-52	0.981141	0.529	0.049	0.48	Venous.EC	*Vcam1*
9.33E-56	1.85E-51	0.773888	0.701	0.1	0.601	Venous.EC	*Thsd7a*
6.20E-54	1.23E-49	1.820038	0.989	0.421	0.568	Venous.EC	*Ehd4*
1.45E-52	2.87E-48	0.599418	0.414	0.026	0.388	Venous.EC	*Lepr*
9.73E-51	1.93E-46	0.473249	0.276	0.007	0.269	Venous.EC	*Fam174b*
3.17E-49	6.28E-45	1.670995	1	0.503	0.497	Venous.EC	*Il6st*
1.75E-48	3.46E-44	1.280101	0.816	0.193	0.623	Venous.EC	*Rasa4*
2.53E-46	5.01E-42	0.499891	0.287	0.01	0.277	Venous.EC	*Enpp6*
3.45E-46	6.82E-42	0.893335	0.471	0.049	0.422	Venous.EC	*Lbp*
4.11E-46	8.12E-42	0.530151	0.402	0.031	0.371	Venous.EC	*Plekha7*
1.36E-42	2.68E-38	1.468284	0.92	0.294	0.626	Venous.EC	*Eln*
2.60E-178	5.15E-174	2.834275	0.941	0.05	0.891	Pericytes	*Vtn*
2.07E-174	4.10E-170	2.736126	0.901	0.041	0.86	Pericytes	*Kcnj8*
3.61E-170	7.15E-166	2.418101	0.954	0.065	0.889	Pericytes	*Pdgfrb*
6.07E-167	1.20E-162	1.855282	0.888	0.039	0.849	Pericytes	*Gucy1a1*
1.72E-166	3.40E-162	1.920828	0.921	0.051	0.87	Pericytes	*Notch3*
1.77E-163	3.51E-159	2.356185	0.934	0.064	0.87	Pericytes	*Steap4*
3.35E-160	6.63E-156	2.506243	0.901	0.059	0.842	Pericytes	*Abcc9*
3.95E-156	7.81E-152	1.723156	0.875	0.049	0.826	Pericytes	*Gucy1b1*
1.68E-155	3.32E-151	1.651181	0.803	0.028	0.775	Pericytes	*Rgs4*
9.01E-154	1.78E-149	1.352332	0.77	0.024	0.746	Pericytes	*Heyl*
2.21E-153	4.37E-149	1.867014	0.855	0.048	0.807	Pericytes	*Higd1b*
2.09E-149	4.14E-145	1.805337	0.882	0.062	0.82	Pericytes	*Cox4i2*
2.05E-143	4.05E-139	2.107985	0.934	0.092	0.842	Pericytes	*Aspn*
1.05E-141	2.07E-137	1.196336	0.77	0.035	0.735	Pericytes	*Arhgef17*
5.20E-134	1.03E-129	2.38312	0.934	0.115	0.819	Pericytes	*Ndufa4l2*
2.23E-133	4.41E-129	1.686806	0.868	0.079	0.789	Pericytes	*Cygb*
2.32E-127	4.58E-123	1.32045	0.822	0.069	0.753	Pericytes	*Lhfp*
2.97E-127	5.88E-123	1.109595	0.664	0.023	0.641	Pericytes	*Itga7*
1.09E-125	2.15E-121	1.188494	0.724	0.037	0.687	Pericytes	*Atp1b2*
7.59E-125	1.50E-120	1.122424	0.724	0.04	0.684	Pericytes	*Cystm1*
